# ARID1A mutation drives gastric tumorigenesis via activating type 2 immune dominant microenvironment

**DOI:** 10.1016/j.isci.2025.113117

**Published:** 2025-07-15

**Authors:** Junya Arai, Yoku Hayakawa, Nobumi Suzuki, Hiroto Kinoshita, Masahiro Hata, Ken Kurokawa, Yuki Matsushita, Sohei Abe, Yukiko Oya, Mayo Tsuboi, Sozaburo Ihara, Yusuke Iwata, Keita Murakami, Toshiro Shiokawa, Chihiro Shiomi, Chie Uekura, Keisuke Yamamoto, Hiroaki Fujiwara, Satoshi Kawamura, Hayato Nakagawa, Tsuneo Ikenoue, Hiroaki Tateno, Tetsuo Ushiku, Hideaki Ijichi, Yoshihiro Hirata, Masato Kasuga, Gloria H. Su, Timothy C. Wang, Mitsuhiro Fujishiro

**Affiliations:** 1 Department of Gastroenterology, Graduate School of Medicine, University of Tokyo, Tokyo, Japan; 2 Division of Gastroenterology, The Institute of Medical Science, Asahi Life Foundation, Tokyo, Japan; 3 Department of Gastroenterology, Graduate School of Medicine, Mie University, Tsu, Mie, Japan; 4 Department of Gastroenterology, Institute of Medical Science, IMSUT Hospital, The University of Tokyo, Tokyo, Japan; 5 Cellular and Molecular Biotechnology Research Institute, National Institute of Advanced Industrial Science and Technology (AIST), Tsukuba, Ibaraki, Japan; 6 Department of Pathology, Graduate School of Medicine, University of Tokyo, Tokyo, Japan; 7 The Institute of Medical Science, Asahi Life Foundation, Tokyo, Japan; 8 Department of Pathology, Columbia University, New York, NY, USA; 9 Division of Digestive and Liver disease, Department of Medicine, Columbia University, New York, NY, USA

**Keywords:** Immunology, Cell biology, Cancer

## Abstract

*ARID1A* is a frequently mutated gene in gastric cancers (GCs), particularly in those associated with the Epstein-Barr virus (EBV), which also often shows *PIK3CA* mutations and *CDKN2A* silencing. However, the role of these alterations in the development of GC remains unclear. Here, using *Tff1*Cre; *Arid1a*^flox/flox^; *Cdkn2a (p16)*^flox/flox^; LSL-*Pik3ca*^*H1047R*^ mice (APP mice), we found that *Arid1a* deletion alone promoted a type 2 immune microenvironment marked by the infiltration of type 2 innate lymphoid cells (ILC2s), eosinophils, mast cells, and M2 macrophages via triggering aberrant IL-33-expressing pit lineage differentiation in stem/progenitor cells. Targeting interleukin (IL)-33, IL-13, and ILC2 activation suppressed metaplasia and tumor progression in APP mice. *Arid1a* and *Pik3ca* mutations cooperatively promoted cell proliferation through v-Akt murine thymoma viral oncogene homolog (AKT) phosphorylation at distinct sites. This type 2 immune response was also observed in human GC samples harboring EBV or *ARID1A* mutations. In conclusion, type 2 immune microenvironment is a hallmark of *ARID1A*-mutated GCs and represents a promising therapeutic target.

## Highlights

• *Arid1a* mutation induced IL-33-positive foveolar hyperplasia through WNT pathway suppression
• IL-33 upregulation activates type 2 immunity in the tumor microenvironment
• Targeting type 2 immunity shows efficacy in *Arid1a*-mutated gastric cancer
• AKT inhibitors are also effective in *Arid1a*-mutated gastric cancers

## Graphical abstract

**Figure:**
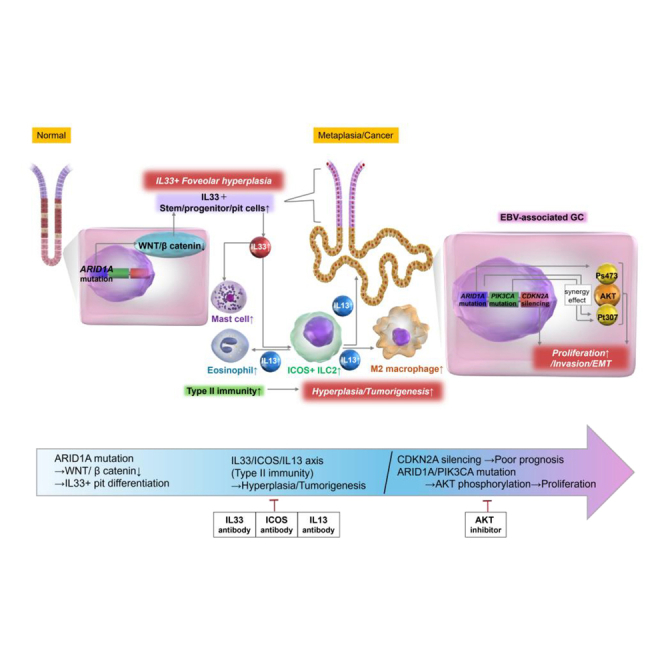

## Introduction

Gastric cancer (GC) is a complex malignancy with diverse genetic profiles and distinct molecular characteristics.^1^ Several studies have revealed that GC can be classified into four genetic subtypes: mismatch repair-deficient tumors carrying high mutation rates, genomically stable tumors associated with the diffuse or signet-ring histological variant, chromosomally unstable tumors displaying significant aneuploidy and focal amplification of receptor tyrosine kinases, and Epstein-Barr virus (EBV)-positive tumors characterized by DNA promoter hypermethylation.^2^^,^^3^^,^^4^^,^^5^

Each subtype has unique genetic mutations; however, some mutations are common across all subtypes. Among common gene mutations, *ARID1A* is one of the most frequent in GC, with a mutation rate of approximately 30%.^2^^,^^4^ The frequency of *ARID1A* mutations in GC subtypes has been reported to be approximately 40%–55% in EBV-positive cases, 40%–80% in microsatellite instability (MSI) cases, and lower in the chromosomal instability (CIN) and genomically stable (GS) subtypes.^2^ ARID1A, also known as BAF250a, encodes a multifunctional subunit of the BRG1/BRM-associated factor (BAF) complex.^6^^,^^7^^,^^8^ It targets the BAF complex to DNA sequences rich in AT base pairs, regulates transcription, and recruits topoisomerase 2 to chromatin. Several previous studies have reported that ARID1A plays an essential role in maintaining cell stemness^9^ and facilitating DNA damage repair.^10^ Hence, *ARID1A* mutations disrupt the BAF complex-mediated chromatin remodeling because this subunit directly interacts with DNA and recruits other transcriptional coactivators. ARID1A’s role as a global regulator of chromatin structure underpins the diverse effects of the disruption of this gene, which complicates the study of its role in cancer development.

Although *ARID1A* mutations are observed in all major GC subtypes, they are most frequently detected in EBV-positive GCs.^11^ EBV-associated GC accounts for approximately 10% of GC cases, predominantly occurs in men, has a relatively early onset, and is located in the upper part of the stomach.^12^ Pathologically, EBV-associated GC typically appears as a moderately-to-poorly differentiated adenocarcinoma with lymphocyte infiltration and a lace-like pattern in the mucosa. However, some EBV-associated GCs resemble conventional adenocarcinomas, which are well-to-moderately differentiated without lymphocyte infiltration or a lace pattern. In addition to *ARID1A* mutations, EBV-associated GCs exhibit a high rate of *PIK3CA* mutations, lack *TP53* mutations, and feature DNA CpG hypermethylation, characterized by *CDKN2A* (*p16*) silencing.^2^ Co-mutations of *ARID1A* and *PIK3CA* are frequently observed,^2^^,^^4^ both of which are associated with poor prognosis.^13^^,^^14^ However, precise carcinogenic mechanisms underlying these genetic changes remain unclear. To explore this, we developed a new mouse model that reflects the genetic profile of human EBV-associated GCs, including *ARID1A* mutations.

## Result

### Arid1a deletion and Pik3ca mutation synergistically induces mucosal hyperplasia and gastric cancer in mice

**Figure 1:**
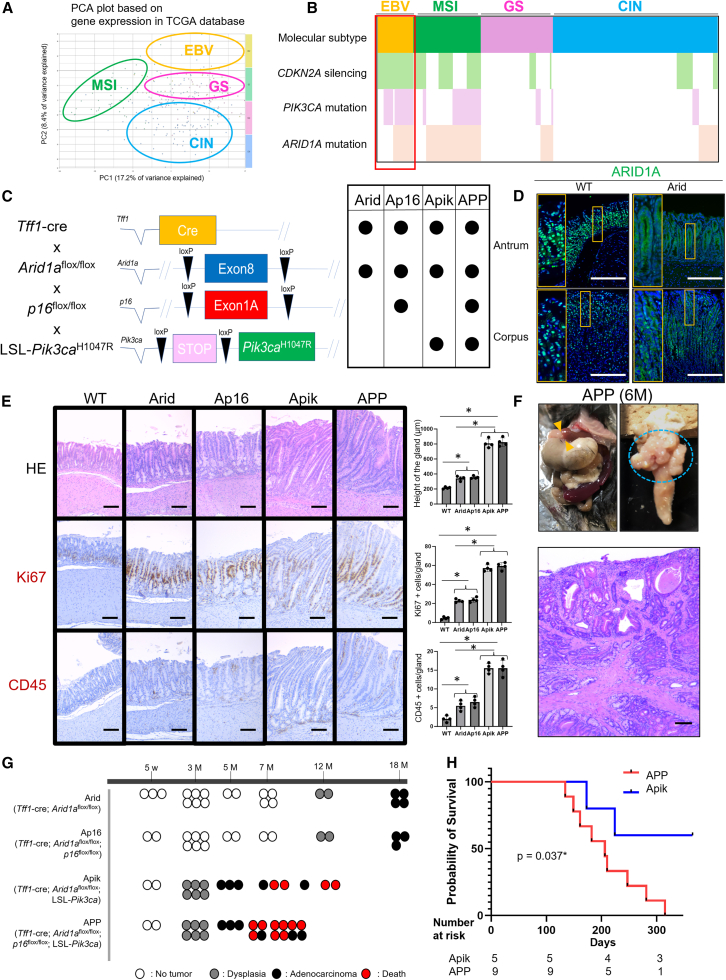
*Arid1a* deletion and *Pik3ca* mutation synergistically induces mucosal hyperplasia and gastric cancer in mice (A and B) Analysis of TCGA database according to the molecular GC subtypes. (A) PCA plot (B) gene mutation map. (C) Construction schema of *Tff1*cre, *Arid1a*^*flox/flox*^, *Cdkn2a (p16)*^*flox/flox*^, and *Loxp-STOP-Loxp Pik3ca*^*H1047R*^ mice. (D) ARID1A staining in WT and *Tff1*cre, *Arid1a*^*flox/flox*^ mice (*n* = 4 mice/group). (E) HE and Ki67/CD45/Alcian blue staining in WT/Arid/Ap16/Apik/APP mice. Numbers of positive cells are quantified (*n* = 4 mice/group). The *p* value was calculated using a t test. (F) Gross findings and HE staining in representative APP mice (aged 6 months) (independent repeats, *n* = 3). (G) Outline mapping of the dysplasia and adenocarcinoma development according to the combination of the *Arid1a*, *Pik3ca*, and *p16* mutations. (H) Cumulative incidence of mortality comparing APP and Apik mice (*n* = 9 and 5 mice/group respectively). The *p* value was calculated using a log rank test. Scale bars, 100 μm. Mean ± standard error of the mean (SEM). ∗*p* < 0.05.

First, we analyzed The Cancer Genome Atlas (TCGA) database^2^ and confirmed that EBV-associated GC showed a high incidence of *ARID1A* and *PIK3CA* mutations along with *CDKN2A* (*p16*) silencing, as described in previous studies (Figures 1A and 1B).^4^^,^^11^ To model these genetic profiles, we developed stomach-specific gene-engineered mice by using *Tff1-*cre, *Arid1a*^*flox/flox*^, *Cdkn2a (p16)*^*flox/flox*^, and LSL-*Pik3ca*^*H1047R*^ mice (Figure 1C). We examined ARID1A expression in the stomachs of wild-type (WT) and *Tff1*-cre; *Arid1a*^*flox/flox*^ mice, in which *Arid1a* was conditionally knocked out in the gastric epithelium (hereafter referred to as Arid mice). ARID1A was broadly expressed in both the antrum and corpus glands in WT mice, whereas its expression was reasonably decreased in Arid mice (Figure 1D). To investigate the interplay between ARID1A, p16, and PIK3CA alterations, we developed 4 genotypes of *Arid1a*-mutated mice: (1) Arid mice, (2) *Tff1-*cre; *Arid1a*^*flox/flox*^; *Cdkn2a (p16)*^*flox/flox*^ (hereafter referred to as Ap16) mice, (3) *Tff1-*cre; *Arid1a*^*flox/flox*^; LSL-*Pik3ca*^*H1047R*^ (Apik) mice, and (4) *Tff1-*cre; *Arid1a*^*flox/flox*^; *Cdkn2a (p16)*^*flox/flox*^; and LSL-*Pik3ca*^*H1047R*^ (APP) mice. Histological analysis revealed that *Arid1a* mutation alone caused mild mucosal hyperproliferation and inflammation in the stomach after 3 months. These hyperplastic and inflammatory changes were significantly accelerated by additional *Pik3ca* mutations (Figures 1E and S1A). In contrast, additional *Cdkn2a* deletion induced no major histological changes (Figures 1E and S1A). We also confirmed that *Tff1-*cre; *Cdkn2a (p16)*^*flox/flox*^ mice showed no obvious changes in the gastric mucosa, whereas *Tff1-*cre; LSL-*Pik3ca*^*H1047R*^ mice showed increased cell proliferation and inflammation (Figures S1B and S1C).

At 6 months of age, APP mice developed macroscopic tumors, mainly located in the antrum, which frequently obstructed the gastric outlet (Figure 1F). Histologically, a massive expansion of highly atypical epithelial cells was observed, accompanied by structural dysplasia as well as robust immune cell infiltration and fibrosis, leading to a diagnosis of adenocarcinoma (Figures 1F and S1D). While Apik and APP mice developed adenocarcinoma in the antrum at the median age of 5 months, Arid and Ap16 mice developed dysplasia and eventually adenocarcinoma in the antrum at approximately 1 and 1.5 years, respectively (Figure 1G). Apik mice exhibited phenotypes similar to APP mice but had a better survival rate (Figure 1H). The phenotypes in the corpus were generally similar to those in the antrum but less pronounced with respect to the increased proliferation and infiltration of inflammatory cells (Figure S2). Overall, our findings suggested that *Arid1a* and *Pik3ca* mutations synergistically contribute to gastric tumorigenesis.

### Aberrant type 2 immunity is involved in Arid1a-mutated tumorigenesis in mice

**Figure 2:**
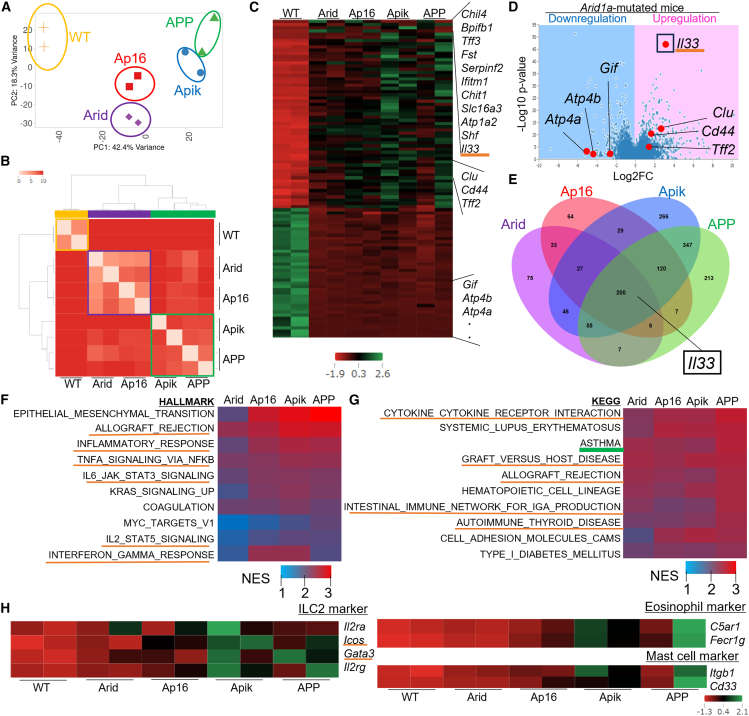
Aberrant type 2 immunity is involved in *Arid1a*-mutated tumorigenesis in mice (A–H) Bulk RNA-seq comparing WT and Arid/Ap16/Apik/APP antrum (*n* = 2 mice/group). (A) PCA plot. (B) Heatmap showing the expression similarity between samples. The distance between samples is measured by calculating the single-parameter quality control and sample comparison for RNA-seq (SERE) coefficient between each pair of samples. (C) Heatmap of the gene expression. (D) Volcano plot. (E) Venn diagram of the upregulated DEGs of Arid/Ap16/Apik/APP mice compared with WT mice. (F) GSEA analysis with Hallmark gene sets shown as heatmap. (G) GSEA analysis with KEGG (Kyoto Encyclopedia of Genes and Genomes) gene sets shown as heatmap. (H) Gene expressions of ILC2, eosinophil, and mast cells shown as heatmap.

Next, we performed a bulk RNA sequencing (RNA-seq) analysis of the gastric antrum in WT, Arid, Ap16, Apik, and APP mice aged 3 months. Therefore, Arid and Ap16 samples included hyperplasia/adenoma, while Apik and APP samples contained intramucosal carcinoma. Principal-component analysis (PCA) and unsupervised clustering indicated that these five mouse strain samples could be broadly divided into three groups based on their transcript levels: (1) WT samples, (2) Arid and Ap16 samples, and (3) Apik and APP samples (Figures 2A and 2B). As highlighted in the heatmap, volcano plot, and Venn diagram, 730 differentially expressed genes (DEGs) were upregulated, including metaplastic markers such as *Clu*, *Cd44*, and *Tff2*, and 416 were downregulated, including genes expressed in normal gastric cell lineages such as *Atp4a*, *Atp4b*, and *Gif* in all *Arid1a*-mutated samples compared with the WT group (Figures 2C–2E; Tables S1 and S2). Notably, *Il33* was dramatically upregulated across *Arid1a*-mutated groups (Table S3).

Gene set enrichment analysis (GSEA) using hallmark gene sets revealed that *Arid1a*-mutated samples were enriched with inflammatory signaling genes mediated by interferon, interleukin (IL)-2, IL-6, or tumor necrosis factor alpha (TNF-α), as well as genes related to various oncogenic pathways, including Kirsten Rat Sarcoma viral oncogene homolog (KRAS) and avian myelocytomatosis viral oncogene homolog (MYC) signaling (Figure 2F; Table S4). Additionally, GSEA using Kyoto Encyclopedia of Genes and Genomes (KEGG) gene sets showed enrichment of multiple gene sets related to inflammation (Figure 2G; Table S5). Among these, we focused on type 2 immunity, as IL-33 is known to be a central mediator of this immune response in the stomach.^15^ The asthma-related gene set, which is closely associated with type 2 immunity, was enriched in all *Arid1a* mutation-associated mice (Figure 2G). Subsequently, inflammatory cell markers involved in type 2 immunity, including type 2 innate lymphoid cells (ILC2s), eosinophils, and mast cells were upregulated in these mice (Figure 2H). We analyzed the RNA-seq data using the CIBERSORTx application,^16^ which confirmed the activation of global inflammatory responses and enrichment of mast cell activation in *Arid1a*-mutated mice (Figures S3A and S3B; Table S6). Immunograms^17^ also indicated greater inflammatory responses in *Arid1a* mutation-associated mice, particularly those with *Pik3ca* mutation (Figure S3C; Table S7). Therefore, *Arid1a*-mutated mice exhibited an immune-rich state in the stomach, with a particular emphasis on type 2 immunity.

### Arid1a mutation disrupts normal differentiation but expands IL-33-expressing pit and metaplastic lineage

**Figure 3:**
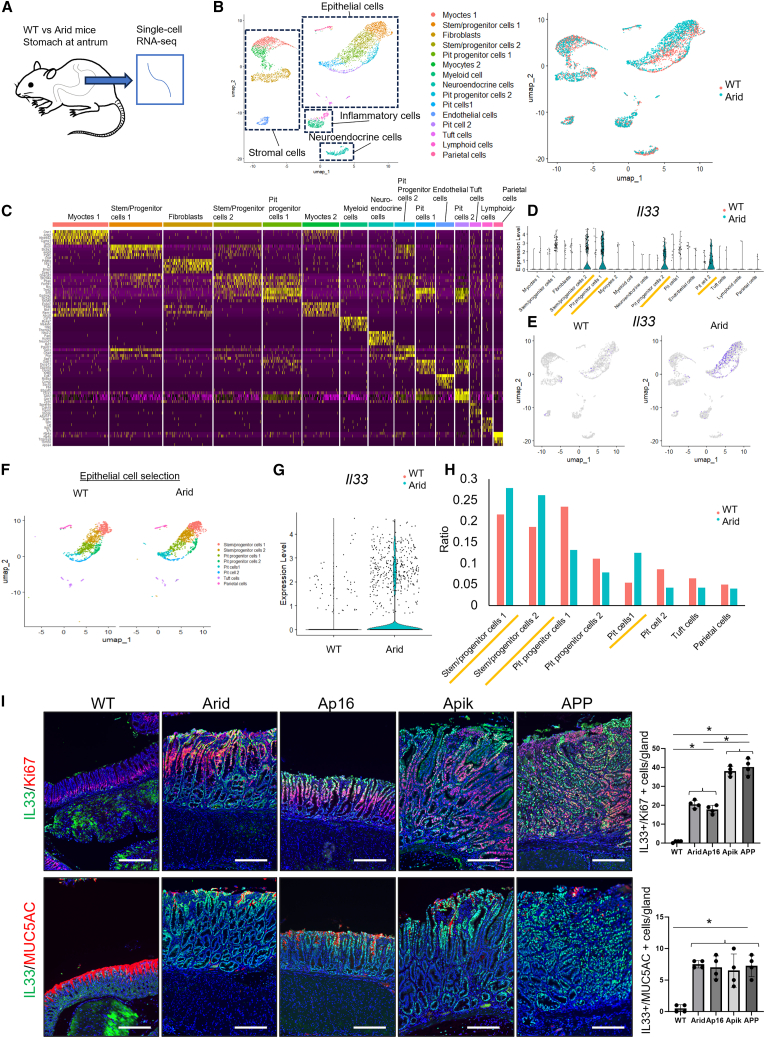
*Arid1a* depletion expands epithelial IL-33 expression in pit cell lineage (A) Schematic representation of the single-cell RNA-seq analysis conducted on gastric specimens from Arid and WT mice. (B) UMAP plot displaying 5,880 cells from the gastric antrum across 15 clusters (left) with cells from Arid and WT mice separately visualized (right). (C) Heatmap showing the top five differentially expressed genes across the 15 clusters. (D) *Il33* expression across the 15 clusters in Arid and WT mice, depicted in a violin plot. (E) Feature plot illustrating *Il33* expression in Arid and WT mice. (F) UMAP plot of gastric epithelial cells in Arid and WT mice. (G) *Il33* expression in gastric epithelial cells from Arid and WT mice, depicted in a violin plot. (H) Distribution of cells across the eight gastric epithelial cell clusters in Arid and WT mice. (I) IL-33 (green)/Ki67 (red) and IL-33 (green)/MUC5AC (red) staining in *Arid1a*-mutated mice. Numbers of positive cells are quantified (*n* = 4 mice/group). The *p* value was calculated using a t test. Scale bars, 100 μm. Mean ± SEM. ∗*p* < 0.05.

To further elucidate the type 2 immunity-dominant microenvironment in *Arid1a*-mutated tissues at the single-cell level, we conducted a single-cell RNA sequencing (scRNA-seq) analysis of WT and Arid mice (Figure 3A). A total of 5,880 cells from the gastric antrum of WT and Arid mice were categorized into 15 clusters, including two pit cell clusters, two pit progenitor (PPG) clusters, two stem/progenitor (SPG) clusters, parietal cells, tuft cells, neuroendocrine cells, myeloid cells, lymphoid cells, two myocyte clusters, fibroblasts, and endothelial cells, based on the DEGs specific to each cluster (Figures 3B, 3C, and S4).

Notably, *Il33* expression was almost exclusively detected in cells from Arid mice, most of which belonged to SPG cells and the pit lineage. (Figures 3D and 3E). This was more evident after selecting and re-analyzing epithelial cell clusters (Figures 3F and 3G). The proportions of SPGs and pit lineage cells were higher in the Arid mice than in the WT mice (Figure 3H). Immunohistochemical staining (IHC) confirmed IL-33 expression in Ki67^+^ proliferating progenitors and MUC5AC^+^ pit cells in *Arid1a*-mutated gastric tissues and tumors, whereas almost no IL-33 expression was observed in the normal gastric antrum (Figure 3I).

**Figure 4:**
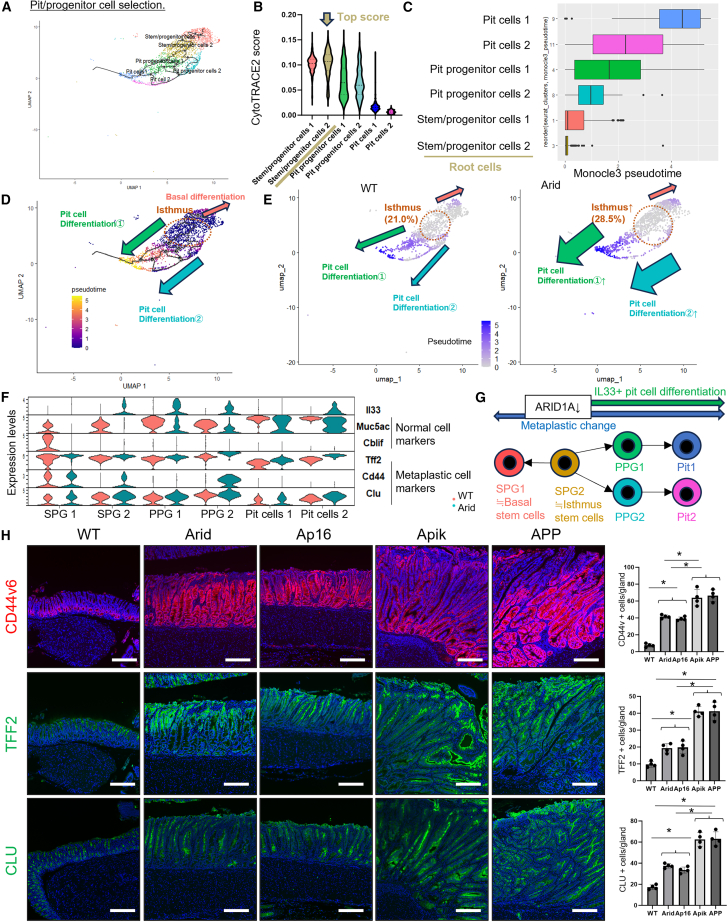
*Arid1a* mutation disrupts normal differentiation but expands IL-33-expressing pit and metaplastic lineage (A) Trajectory analysis as visualized in the UMAP plot depicting gastric pit/pit progenitor/progenitor cells in WT and Arid mice. (B) Calculation of CytoTRACE2 scores according to the cell clusters presented in a violin plot. (C) Ordering of cell clusters based on Monocle3 pseudotime analysis. (D) Pseudotime calculation visualized in the UMAP plot. (E) Pseudotime calculation in the UMAP plot, separated by WT and Arid mice. (F) Expression of *Il33*, *Muc5ac*, *Cblif*, *Tff2*, *Cd44*, and *Clu* according to the cell clusters as visualized in violin plot. (G) The scheme illustrating alterations in cell differentiation induced by the *Arid1a* mutation. (H) CD44v6, TFF2, and CLU staining in *Arid1a*-mutated mice. Numbers of positive cells are quantified (*n* = 4 mice/group). The *p* value was calculated using a t test. Scale bars, 100 μm. Mean ± SEM. ∗*p* < 0.05.

Next, we elucidated the mechanism by which *Arid1a* mutations induce IL-33-expressing foveolar (pit cell) hyperplasia. We performed a pseudotime trajectory analysis of scRNA-seq data to elucidate the impact of the *Arid1a* mutation on cell differentiation within pit cells, PPGs, and SPGs (Figure 4A). Based on the results of the trajectory analysis and Cytotrace2 scores (Figures 4A and 4B), the SPG 2 cluster was defined as the root cell for pseudotime calculation (Figure 4C). Pseudotime analysis revealed three main differentiation pathways: (1) SPG2 to PPG1 to pit cell 1, (2) SPG 2 to PPG 2 to pit cell 2, and (3) SPG 2 to SPG 1. Consequently, SPG 2 was considered an ithmus stem cell in the antrum, whereas SPG 1 was considered a basal stem cell^18^^,^^19^ (Figure 4D). In the *Arid1a*-mutant mice, the population of isthmal SPG 2 cells was greater and the differentiation into the pit cell lineage was higher than in WT mice (Figure 4E).

In terms of gene expression profile, *Il33* is reported to be expressed in a subset of normal pit cells.^15^^,^^20^ However, in Arid mice, *Il33* was highly expressed even in more undifferentiated SPG 2 and PPG 1/2 clusters, in addition to mature pit clusters, such as pit cell 2 (Figure 4F), suggesting that dysregulated IL-33 expression occurs from stem/progenitor cell states by *Arid1a* mutation. In contrast, markers for mature differentiation, including *Cblif* and *Muc5ac*, were downregulated in the basal and pit clusters in Arid mice. In addition, markers of gastric metaplasia, including *Tff2*, *Cd44*, and *Clu*, were upregulated in *Arid1a*-mutated SPGs, PPGs, and even mature pit cell clusters (Figures 4F, 4G, and S5). Immunostaining confirmed an increase in metaplastic markers and a decrease in differentiation markers in *Arid1a*-mutated mice (Figures 4H and S6). These findings suggest that the *Arid1a* mutation disrupts normal differentiation of isthmus stem/progenitor cells, resulting in the expansion of undifferentiated stem/progenitor cells, metaplasia, and IL-33-expressing pit lineage.

### Expansion of IL-33-expressing lineage is driven by WNT inactivation mediated through Arid1a mutation

**Figure 5:**
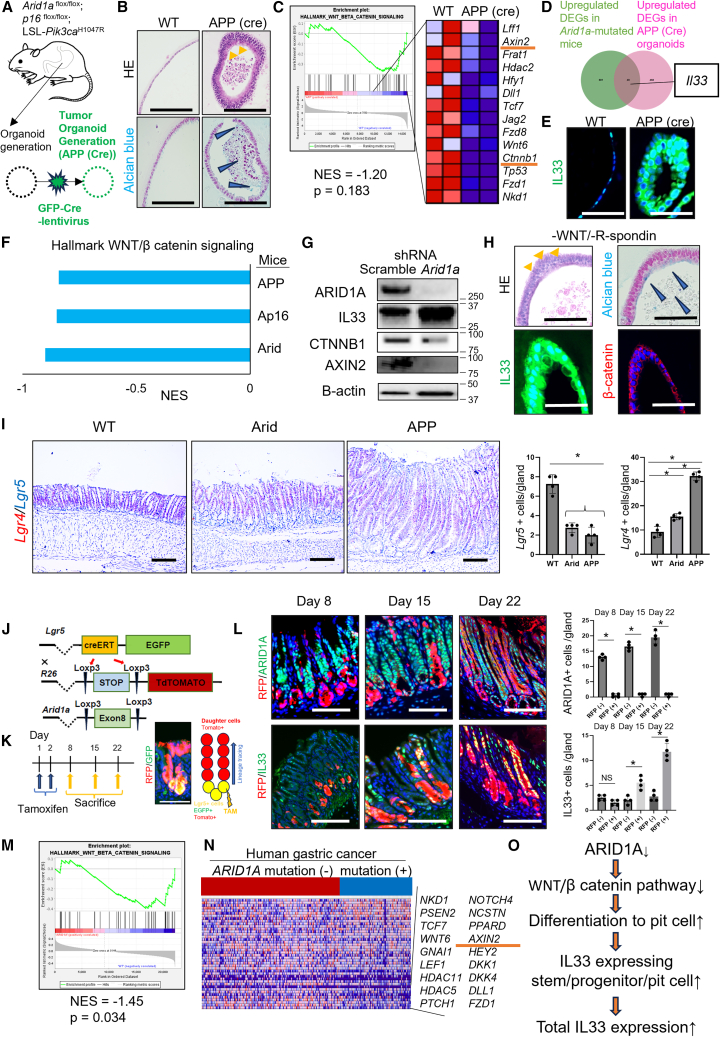
Expansion of IL-33-expressing lineage is driven by WNT inactivation mediated through *Arid1a* mutation (A) Schematic representation of tumor organoid generation. Organoids generated from *Arid1a*^*flox/flox*^, *Cdkn2a (p16)*^*flox/flox*^, and *Loxp-STOP-Loxp Pik3ca*^*H1047R*^ mice were infected with GFP-Cre lentivirus (hereafter APP [Cre] organoids). (B) HE and Alcian blue staining in WT and APP (Cre) organoids (*n* = 20/group). (C) GSEA with gene sets of WNT/β-catenin pathway in WT and APP (Cre) organoids. (D) Venn diagram of the upregulated DEGs of APP (Cre) organoids and *Arid1a-*mutated mice. (E) IL-33 staining in WT and APP (Cre) organoids (*n* = 20/group). (F) GSEA with gene sets of WNT/β-catenin pathway in Arid, Ap16, and APP mice compared with WT mice. (G) WT organoids were treated with shRNA-*Arid1a* or a control adenovirus. Western blots for ARID1A, IL-33, CTNNB1, AXIN2, and β-actin are shown (independent repeats: *n* = 3). (H) WT organoids were cultured in a medium without WNT/R-spondin. HE, Alcian blue, MUC5AC (red)/IL-33 (green), and β-catenin (red) staining are shown (*n* = 20/group). (I) RNA-ISH using Lgr4 (red)/Lgr5 (blue) probes in WT, Arid, and APP mice (*n* = 4/group). The *p* value was calculated using a t test. (J–L) Lineage tracing experiments. (J) Gene construction schema of *Lgr5*-CreERT; *Rosa26*-Lox-Stop-Lox-*tdTomato*; *Arid1a*^*flox/flox*^ mice. (K) Schematic of the experimental protocol and representative image of tdTomato and GFP expression in the antrum gland following tamoxifen induction. (L) RFP (red)/ARID1A (green) (top) and RFP (red)/IL-33 (green) (bottom) staining of *Lgr5*-CreERT; *Rosa26*-Lox-Stop-Lox-*tdTomato*; *Arid1a*^*flox/flox*^ mice (days 8, 15, and 22). Numbers of ARID1A and IL-33-positive cells in the RFP-positive cells and RFP-negative cells are quantified (*n* = 4/group). The *p* value was calculated using a t test. (M and N) GSEA with gene sets of WNT/β-catenin pathway in human gastric cancers with and without *ARID1A* mutation. (M) Sigmoid curve. (N) Heatmap of representative WNT target genes. (O) Schema of the relationship between *ARID1A* mutation, WNT/β-catenin pathway, and IL-33 upregulation. Scale bars, 100 μm. Mean ± SEM. ∗*p* < 0.05.

To investigate the mechanism underlying these findings, we generated organoids from mice harboring the same gene mutations, and then infected the organoids with a lentivirus expressing Cre to develop APP (Cre) organoids (Figure 5A). We confirmed the deletion of *Arid1a* and *p16* in APP (Cre) organoids using IHC and quantitative PCR (Figures S7A and S7B). In previous studies with human stomach organoids, *Arid1a* mutation inactivated the wingless and integration 1 (WNT)/β-catenin pathway, which promotes differentiation toward surface pit cells with loss of polarity and mucinous changes.^21^^,^^22^^,^^23^ In line with this result, RNA-seq data using WT and APP (Cre) organoids revealed that the gene set of the WNT/β-catenin pathway, which is characterized by *Ctnnb1* and *Axin2*, was downregulated in APP (Cre) organoids compared with the WT, accompanied by polarity loss and mucinous changes (Figures 5B and 5C). Notably, *Il33* was upregulated in APP (Cre) organoids (Figures 5D; Tables S9 and S10). IHC revealed an increase in IL-33 positive cells in the APP organoids (Figure 5E). *Arid1a-*mutated stomachs, including Arid, Ap16, and APP, showed similar trends in terms of the downregulation of WNT/β-catenin pathway in bulk RNA-seq (Figure 5F).

Next, we treated WT organoids with *Arid1a*-shRNA-expressing adenoviral vectors to examine the effects of *Arid1a* knockdown. *Arid1a* knockdown induced increased IL-33 expression and decreased the expression of AXIN2 and β-catenin (Figure 5G). WT organoids cultured in a medium lacking WNT/R-spondin exhibited a loss of polarity, mucinous changes stained with Alcian blue, and upregulation of IL-33 (Figure 5H). We also confirmed the decreased expression of *Lgr5*, a major WNT target in the gastric antrum, by in situ hybridazation (ISH) in Arid and APP mice (Figure 5I).

We then investigated the role of *Arid1a* in SPGs in *Lgr5*-CreERT; *Arid1a*^flox/flox^; *Rosa26*-LSL-*tdTomato* mice using lineage-tracing experiments and selective gene depletion. After tamoxifen induction, ARID1A expression was efficiently depleted in the *tdTomato*-expressing *Lgr5*-derived antral glands over time. Interestingly, at earlier time points, IL-33 expression was observed in *Arid1a*-depleted *tdTomato*-expressing basal cells, and these IL-33^+^ clones gradually expanded and eventually occupied the entire gland (Figures 5J–5L).

TCGA database showed that human GCs with *ARID1A* mutation is associated with the downregulation of the gene set of the WNT/β-catenin pathway compared with GCs without *ARID1A* mutation, and the expression of many WNT target genes such as AXIN2 was significantly decreased (Figures 5M and 5N). Therefore, data from mouse organoids and tissues, as well as human GC database, suggest that *Arid1a* mutation suppresses WNT/β-catenin signaling, which leads to the expansion of the IL-33-expressing lineage (Figure 5O).

### Activated IL-33/IL-13 axis could be a therapeutic target in Arid1a-mutated gastric cancers

**Figure 6:**
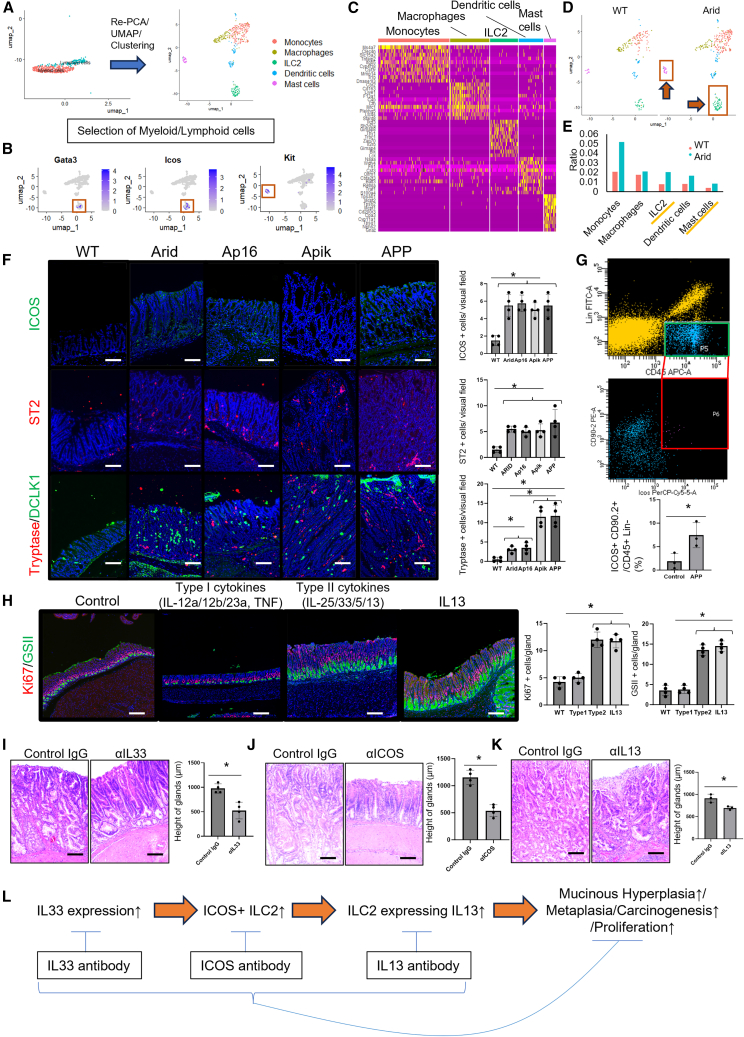
Activated IL-33/IL-13 axis could be a therapeutic target in *Arid1a*-mutated gastric cancers (A) Re-PCA, UMAP, and the clustering of inflammatory cells are shown. (B) Feature plots of *Gata3*, *Icos*, and *Kit* shown as UMAP plots. (C) Heatmap showing the top 10 differentially expressed genes across the five clusters. (D) Five clusters split by WT and Arid mice shown as UMAP plots. (E) Distribution of cells across the five inflammatory cell clusters in Arid and WT mice. (F) ICOS (green), ST2 (red), and tryptase (red)/DCLK1 (green) staining of WT and Arid/Ap16/Apik/APP mice. Numbers of positive cells per field are quantified (*n* = 4/group). The *p* value was calculated using a t test. (G) FACS gating strategy of ILC2s. CD45^+^Lin^−^CD90.2^+^ICOS^+^ cells were defined as ILC2s and the numbers in WT and APP mice were quantified (*n* = 3/group). The *p* value was calculated using a t test. (H) Treatment with adeno-associated virus vectors expressing type 1 cytokines (IL-12a/-12b/-23a and TNF), type 2 cytokines (IL-25/-33/-5/-13), and IL-13 in WT mice. Ki67 (red)/GSII (green) staining is shown. Numbers of positive cells are quantified (*n* = 4 mice/group). The *p* value was calculated using a t test. (I) H&E staining of APP mice treated with control antibody or neutralizing antibody against IL-33. Average gland heights were quantified (*n* = 4/group). The *p* value was calculated using a t test. (J) H&E staining of APP mice treated with control antibody or neutralizing antibodies against ICOS. Average gland heights were quantified (*n* = 4/group). The *p* value was calculated using a t test. (K) H&E staining of APP mice treated with control antibody or neutralizing antibody against IL-13. Average gland heights were quantified (*n* = 3/group). The *p* value was calculated using a t test. (L) Scheme of the type 2 immunity-dependent tumorigenesis in APP mice. Scale bars, 100 μm. Mean ± SEM. ∗*p* < 0.05.

We focused on immune cell clusters, particularly the myeloid and lymphoid cell clusters, within the scRNA-seq dataset (Figure 6A). In total, 505 cells were classified into five clusters based on their respective cell markers: monocytes, macrophages, ILC2s, dendritic cells, and mast cells (Figures 6B and 6C). Arid mice exhibited an increase in most inflammatory cell types, including ILC2s and mast cells, both of which are involved in type 2 immunity (Figures 6D and 6E). IHC confirmed the presence of these cell types. *Arid1a*-mutated mouse lines exhibited robust infiltration of inducible T cell costimulator (ICOS)/growth stimulation expressed gene 2 (ST2)-positive ILC2s, direct fast scarlet (DFS)-positive eosinophils, CD206-positive M2 macrophages, tryptase-positive mast cells, and doublecortin-like kinase 1 (DCLK1)-positive tuft cells, all of which play central roles in type 2 immunity in the stomach (Figures 6F and S8).^24^^,^^25^^,^^26^^,^^27^^,^^28^^,^^29^^,^^30^^,^^31^ We further validated the increased ILC2s (defined as CD45^+^Lin^−^ICOS^+^CD90.2^+^ cells) using fluorescence-assisted cell sorting (FACS) (Figure 6G). Next, we examined the effect of type 2 cytokines, such as IL-25, IL-33, IL-5, and IL-13, on gastric mucosal changes by treating mice with adeno-associated virus vectors. Treatment with type 2 cytokine-expressing vectors, as well as vectors expressing IL-13 alone, induced increased proliferation and mucous hyperplasia, as observed in *Arid1a*-mutated mice (Figure 6H). In contrast, treatment with type 1 cytokine-expressing vectors did not induce this phenotype. Thus, the activated type 2 immune microenvironment is involved in the aberrant proliferation and differentiation of the *Arid1a*-mutated gastric epithelium.

When mice were infected with *Helicobacter pylori* (PMSS-1 strain), Arid mice exhibited more robust proliferation and mucinous changes than control mice, along with IL-33 overexpression in the gastric epithelium and increased infiltration of ICOS-positive ILC2s (Figure S9). We previously reported that mutations in *Pten*, *Kras*, and *Cdh1* in the gastric epithelium induce various types of metaplasia.^32^ When Arid mice were mated with *Pten*^flox/flox^ mice, macroscopic tumors developed both in the antrum and corpus, and these tumors histologically resembled well-differentiated human intestinal-type GCs (Figure S10). The combination of *Arid1a* deletion with *Kras* or *Cdh1* mutations led to the development of a highly proliferative mucous-rich metaplasia (Figure S11). Given that IL-33 expression and infiltration of ILC2s are evident in these tumors and metaplasia, *Arid1a*-dependent type 2 immune microenvironment appears to play a role in various settings of gastric tumorigenesis.

Next, we targeted the type 2 immune microenvironment in *Arid1a*-mutated GC. First, we treated APP mice with a neutralizing antibody against IL-33, which eventually reduced the tumor size and number of proliferating cells and ICOS-positive ILC2s within the tumors (Figures 6I and S12A–S12C). When APP mice were treated with a neutralizing antibody against ICOS for ILC2 depletion, they exhibited smaller tumors with less proliferation and metaplasia (Figures 6J and S12D–S12F). We also confirmed that treatment with a neutralizing antibody against IL-13 led to reduced tumor size, proliferation, and metaplasia in APP mice (Figures 6K and S12G–S12I). Therefore, the IL-33/IL-13 axis may be a potential therapeutic target for *Arid1a*-mutated GCs, particularly for the early or precancerous lesions (Figure 6L).

### ARID1A and PIK3CA cooperatively orchestrate AKT phosphorylation and cell proliferation in gastric epithelial cells

**Figure 7:**
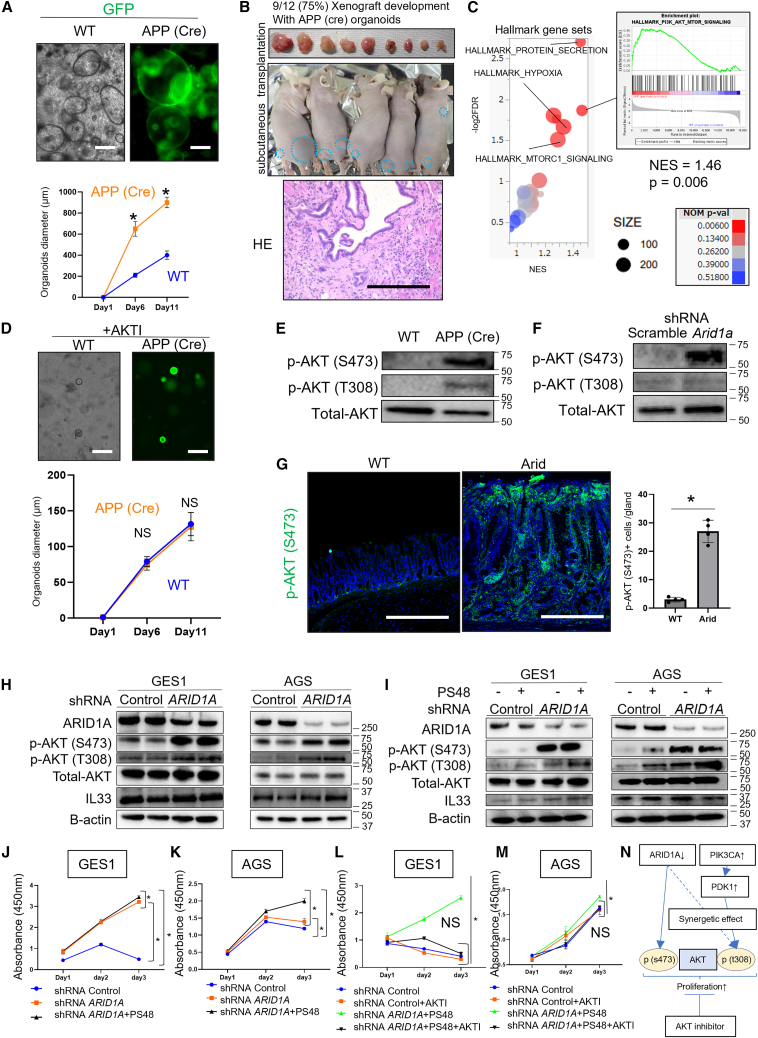
ARID1A and PIK3CA cooperatively orchestrate AKT phosphorylation and cell proliferation in gastric epithelial cells (A) Representative image and proliferation assay of WT and APP (Cre) organoids. Organoid diameters were quantified at the indicated points (*n* = 20/group). The *p* value was calculated using a t test. (B) Gross findings in APP (Cre) xenografts. The success rate of xenograft generation was 9/12 (75%). A representative HE image is shown. (C) GSEA of hallmark gene sets with bulk RNA-seq for WT and APP (Cre) organoids is shown as a bubble plot. (D) Treatment with AKT inhibitor (MK-2206) in WT and APP (Cre) organoids. Organoid diameters were quantified (*n* = 20/group). The *p* value was calculated using a t test. (E) Western blotting of *p*-AKT (S473), *p*-AKT (T308), and total-AKT in WT and APP (Cre) organoids is shown (independent repeats: *n* = 3). (F) WT organoids were treated with shRNA-*Arid1a* or a control adenovirus. Western blots of *p*-AKT (S473), *p*-AKT (T308), and total-AKT are shown (independent repeats: *n* = 3). (G) *p*-AKT (Ser473) staining in WT and Arid mice. Numbers of positive cells are quantified (*n* = 4/per group). The *p* value was calculated using a t test. (H) GES1 and AGS cell lines were treated with control or shRNA-*ARID1A* adenovirus. Western blots of ARID1A, *p*-AKT (S473), *p*-AKT (T308), total-AKT, IL-33 and β-actin are shown (independent repeats: *n* = 2). (I) GES1 and AGS cell lines were treated with the control or shRNA-*ARID1A* adenovirus, with or without PS48. Western blots of ARID1A, *p*-AKT (S473), *p*-AKT (T308), total-AKT, IL-33 and β-actin are shown (independent repeats: *n* = 2). (J) GES1 cells were treated with the control, shRNA-*ARID1A* adenovirus, or PS48. The cell proliferation assay is shown (*n* = 5/group). The *p* value was calculated using a t test. (K) AGS cells were treated with the control, shRNA-*ARID1A* adenovirus, or PS48. The cell proliferation assay is shown (*n* = 5/group). The *p* value was calculated using a t test. (L) GES1 cells were treated with control, shRNA-*ARID1A* adenovirus, PS48, or AKT inhibitors (MK-2206). The cell proliferation assay is shown (*n* = 5/group). The *p* value was calculated using a t test. (M) AGS cells were treated with control, shRNA-*ARID1A* adenovirus, PS48, or AKT inhibitors (MK-2206). The cell proliferation assay is shown (*n* = 5/group). The *p* value was calculated using a t test. (N) Schema of the relationship among ARID1A, PIK3CA, and AKT phosphorylation. Scale bars, 100 μm. Mean ± SEM. ∗*p* < 0.05.

Next, we analyzed the APP (Cre) organoids to evaluate cell-intrinsic pathways. APP (Cre) organoids showed more rapid growth than WT organoids (Figure 7A). APP (Cre) organoids showed partial niche independence, as they could grow in the absence of epidermal growth factor (EGF) and fibroblast growth factor (FGF) (Figure S13A). APP (Cre) organoids were transplanted as subcutaneous xenografts into nude mice (75% development) (Figure 7B). IHC revealed increased IL-33-positive epithelial cells, increased Alcian blue/GSII-positive mucous cells, and apparent infiltration of inflammatory cells involved in type 2 immunity in the APP (Cre) xenografts (Figure S13B), compatible with *Arid1a*-mutated stomach. Based on GSEA of bulk RNA-seq, certain oncogenic pathways were significantly enriched, including the PI3K/v-Akt murine thymoma viral oncogene homolog (AKT) pathway, in the APP (Cre) organoids (Figure 7C). A specific Akt inhibitor, MK-2206, abolished the growth advantage in APP (Cre) organoids, suggesting the critical involvement of the PI3K/AKT pathway in the cell growth of these organoids (Figure 7D). AKT was phosphorylated at both Ser473 and Thr308 in the APP (Cre) organoids (Figure 7E). In contrast, organoids with a specific *Arid1a* knockdown showed AKT phosphorylation only at Ser473 but not at Thr308 (Figure 7F). The stomach mucosa of Arid mice also showed an increase in *p*-AKT (Ser473)-positive epithelial cells (Figure 7G). In contrast, IL-33 treatment of WT organoids did not induce AKT phosphorylation or enhance proliferation (Figures S13C and S13D). Thus, our data suggest that *Arid1a* deletion induces AKT phosphorylation at Ser473, whereas AKT phosphorylation at Thr308 is regulated by *Pik3Ca* mutation.

Next, we used the human cell lines, GES1 and human gastric adenocarcinoma cell line (AGS), which represent normal and GC cells, respectively. *ARID1A* knockdown using shRNA-*ARID1A* induced AKT phosphorylation at Ser473 in both cell lines, and AGS cells also showed AKT phosphorylation at Thr308 without a clear change of IL-33 expression (Figure 7H). Next, we treated these cell lines with PS48, which activates PDK1,^33^^,^^34^ to mimic the effect of PIK3CA activation, and evaluated AKT phosphorylation. PS48 treatment induced AKT phosphorylation, particularly at Thr308, in both cell lines, whereas only minimal changes occurred in Ser473 phosphorylation (Figure 7I). PS48-dependent Thr308 phosphorylation was more evident in cells treated with shRNA-*ARID1A* vectors (Figure 7I). *ARID1A* knockdown alone promoted the growth of GES1 and AGS cells, whereas PS48 treatment enhanced this growth-promoting effect (Figures 7J and 7K). Interestingly, the AKT inhibitor MK-2206 blocked the growth-promoting effects of *ARID1A* knockdown and PS48 treatment, suggesting a central role for AKT activation in cell proliferation (Figures 7L and 7M). Therefore, the cooperative interaction between ARID1A, PIK3CA, and AKT contributes to the proliferation of mouse and human gastric epithelial cells (Figure 7N).

### Type 2 immunity activation is conserved in human EBV-associated or ARID1A-mutated gastric cancer

**Figure 8:**
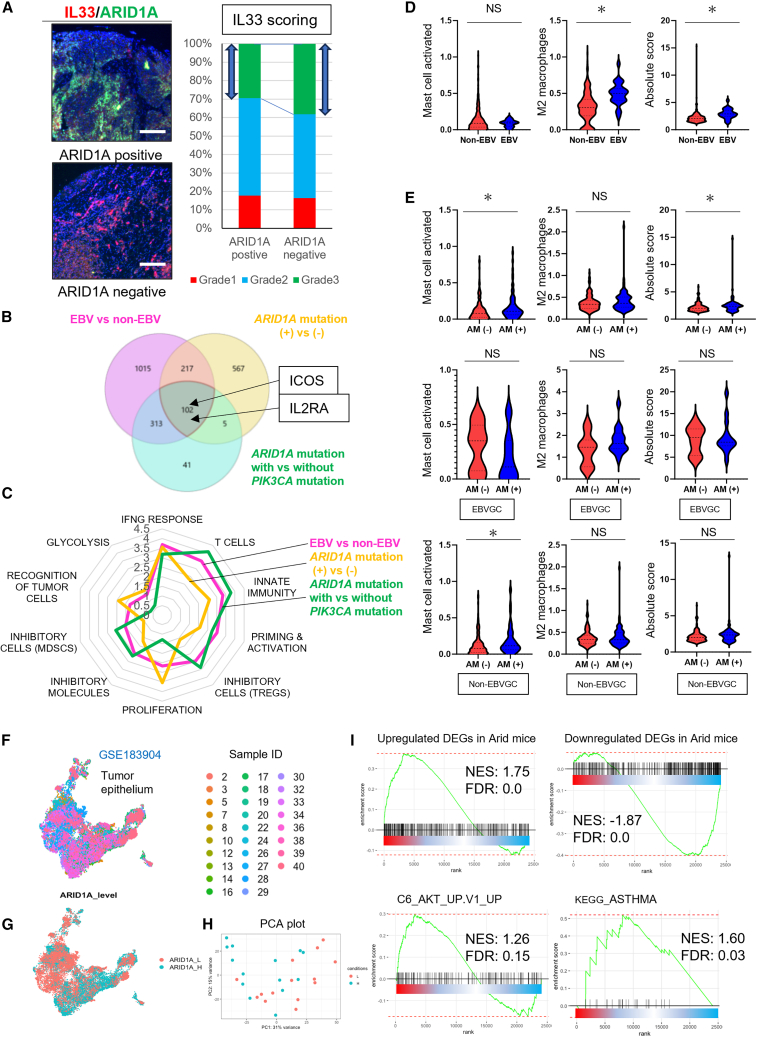
Type 2 immunity activation is conserved in human Epstein-Barr virus-associated or *ARID1A*-mutated gastric cancer (A) Analyzing human tissue array with ARID1A-positive (*n* = 17) and -negative (*n* = 55) GC. Representative ARID1A (green)/IL-33 (red) staining is shown. The grade of IL-33 staining was scored. (B and C) Comparison GCs between Epstein-Barr virus (EBV)-associated and non-associated cases (*n* = 24 vs. 241), as well as mutated and non-mutated *ARID1A* cases in total GCs (*n* = 84 vs. 176) and mutated and non-mutated *PIK3CA* cases with *ARID1A* mutation (*n* = 37 vs. 47), from The Cancer Genome Atlas data. (B) A Venn diagram shows the common upregulated DEGs in EBV-associated, *ARID1A*-mutated, and *ARID1A/PIK3CA*-mutated GCs. (C) Immunogram analysis results are presented as a radar diagram. (D and E) Values of mast cells (left), M2 macrophages (middle), and absolute scores (right) analyzed with CIBERSORTx comparing GCs between (D) EBV-associated and non-associated cases in total GCs (*n* = 24 vs. 241) and (E) *ARID1A*-mutated (AM[+]) and non-mutated (AM[−]) cases in total GCs (top) (*n* = 84 vs. 176), EBV-associated (middle) (*n* = 14 vs. 10), and non EBV-associated GCs (bottom) (*n* = 70 vs. 166), shown as a violin plot. The *p* value was calculated using a t test. (F–I) The analysis of public single-cell RNA-seq data for GC (GSE183904) comparing tumor epithelial samples with high *ARID1A* expression to those with low *ARID1A* expression. (F) UMAP plot colored by GC patient IDs. UMAP plot (G) and PCA plot (H) stratified on the bases of *ARID1A* expression. (I) GSEA with upregulated and downregulated DEGs in ARID mice and gene sets of PI3K/AKT pathway and asthma. Scale bars, 100 μm. Mean ± SEM. ∗*p* < 0.05.

We analyzed a tissue array containing 72 GC cases (Lauren’s intestinal-type cancers) and found a tendency toward higher IL-33 expression in ARID1A-negative cases than in ARID1A-positive cases (Figure 8A). Next, we analyzed bulk transcriptomic data from TCGA and identified upregulated DEGs in EBV-associated GCs compared with non-EBV-associated GCs, *ARID1A*-mutated GCs compared with *ARID1A*-nonmutated GCs, and *ARID1A* and *PIK3CA* mutated GCs compared with *ARID1A* mutated GCs without *PIK3CA* mutations. We found that ILC2 markers, including ICOS and IL2RA, were upregulated in EBV-associated GCs, *ARID1A*-mutated GCs, and *ARID1A* and *PIK3CA* mutated GCs (Figures 8B and S14A–S14C). Furthermore, immunogram analysis revealed an immune-rich state of these GCs (Figures 8C; Table S11).

GSEA using hallmark gene sets revealed that EBV-associated or *ARID1A*-mutated GCs were enriched with inflammatory signaling genes mediated by interferon or IL-6, as well as genes related to various oncogenic pathways, including MYC signaling (Figure S14D; Table S12). GSEA using KEGG gene sets also identified enrichment of multiple genes related to inflammation, especially the asthma gene set (Figure S14D; Table S12). Next, we analyzed the data using the CIBERSORTx application, which indicated activated states of total inflammation, M2 macrophages, and mast cells in EBV-associated or *ARID1A*-mutated GCs (Figures 8D and 8E). The absolute score was the sum of the individual immune cell scores. We also analyzed public scRNA-seq data for GC (GSE183904)^35^ to confirm the phenotype observed in Arid mice within the context of human *ARID1A*-mutated GC. The gene set consisting of upregulated DEGs in Arid mice was enriched in GC cells with low *ARID1A* expression compared with cells with high *ARID1A* expression. Conversely, the gene set of downregulated DEGs in ARID mice was enriched in GC cells with high ARID1A expression levels. In addition, gene sets related to the PI3K/AKT pathway and asthma were enriched in *ARID1A*-low cells (Figures 8F–8I).

Finally, we validated the relationship between IL-33 expression and type 2 immune response. TCGA database showed upregulation of ILC2 markers, including ICOS and GATA3, in GC cases with high IL-33 expression (Figure S14E). Public scRNA-seq data for GC (GSE183904)^35^ showed increased infiltration of mast cells in GC cases with higher IL-33 expression (Figures S14F–S14H). Taken together, in real-world GC cases accompanied by EBV infection or *ARID1A* mutation in cooperation with *PIK3CA* mutation, the type 2 immune microenvironment and PI3K/AKT activation are likely to be conserved and may be potential therapeutic targets.

## Discussion

Our study demonstrates that *Arid1a* deletion, in conjunction with *Pik3ca* mutation, leads to the spontaneous development of mucinous gastritis, hyperplasia, and tumorigenesis in mouse models. *Arid1a* deletion shapes the type 2 immune microenvironment to recruit unique inflammatory mediators, which not only promote tumorigenesis but also serve as a therapeutic target.

Our murine models harboring specific gene mutations characteristic of human EBV-associated GCs did not completely mimic the histology of EBV-associated GCs in humans, which typically manifest as moderately to-poorly differentiated adenocarcinomas with lymphocytic infiltration. This histopathological difference may be attributed to the presence or absence of EBV infection. EBV infection is known to modulate immune responses, including the activation of immune checkpoints and the recruitment of immune cells, which could influence the tumor microenvironment.^36^ To better understand the contribution of EBV infection itself, future studies could involve the use of EBV-infected mouse models or transgenic mice expressing EBV proteins to investigate how viral factors influence immune cell infiltration, tumor progression, and the immune landscape in GC. This approach would help clarify the interplay between genetic mutations and viral infection in the development of EBV-associated GC and enhance the translational relevance of our findings.

Co-occurrence of *ARID1A* and *PIK3CA* mutations are frequently observed in various cancers.^37^^,^^38^^,^^39^^,^^40^ In gastric cell lines, we found that *Arid1a* mutation predominantly induces AKT phosphorylation at Ser473, whereas AKT phosphorylation at Thr308 may require both Arid1a inhibition and PI3K activation. These findings suggest that AKT inhibitors can potentially exert a powerful suppressive effect on human GCs harboring *ARID1A* and *PIK3CA* mutations.^41^

In mice and organoids, IL-33 upregulation occurs via inactivation of the WNT/β-catenin pathway induced by Arid1a downregulation alone. However, ARID1A knockdown in human cell lines did not increase IL-33 expression. This discrepancy might be due to the possibility that IL-33 upregulation driven by ARID1A deletion results from altered differentiation rather than cell-intrinsic mechanisms, given that established cell lines do not recapitulate pit cell differentiation. However, further studies with human gastric epithelial cells or patient-derived organoids/xenografts are required to clarify the relationship between ARID1A and IL-33 expression.

IL-33 overexpression is a distinguishing feature in GC models with *Arid1a* mutation, as such dramatic upregulation is not observed in other GC models lacking *Arid1a* mutation, such as those with mutations in *Kras*, *Cdh1*, *Apc*, or *Muc6*.^32^^,^^42^^,^^43^ Furthermore, although many murine GC models exhibit significant inflammation, *Arid1a-*mutated mice specifically show marked infiltration of type 2 immunity-associated inflammatory cells alongside pronounced mucinous changes in the current study. This suggests that the IL-33-driven, type 2 immunity-dominant microenvironment may be a unique characteristic of *Arid1a*-mutant GCs.

The cytokines IL-33 and IL-13, central players in type 2 immune responses, play significant roles in promoting an immunosuppressive tumor microenvironment that favors cancer progression. In GCs, elevated IL-33 and IL-13 levels have been linked to enhanced tumor-associated macrophage activation, which promotes immune evasion.^25^^,^^44^ These cytokines also drive the differentiation of M2 macrophages, which suppress T cell responses and support tumor growth.^45^ IL-33 and IL-13 have been implicated in the progression of other cancers, such as breast and lung cancers.^46^^,^^47^ Furthermore, the IL-33/IL-13 axis has been shown to elevate PD-L1 expression, thereby inhibiting T cell activation and fostering a suppressive immune microenvironment.^48^^,^^49^ Thus, IL-33 and IL-13 may not only contribute to inflammation but also create a conducive environment for tumor progression by inhibiting antitumor responses.

Several medications have been developed and are currently being studied in clinical trials for other diseases, with potential clinical applications in targeted therapy against type 2 immunity. Itepekimab, a monoclonal antibody targeting IL-33,^50^ dupilumab, a humanized IgG4 monoclonal antibody that targets the IL-4 receptor alpha chain (IL-4Rα)—which is common to both IL-4 receptor complexes: type 1 (IL-4Rα/γc; specific to IL-4) and type 2 (IL-4Rα/IL-13Rα1; specific to IL-4 and IL-13),^51^ and tralokinumab, a human monoclonal antibody to IL-13^52^ are primarily used for inflammatory diseases. These medications may serve as potential candidates for *ARID1A*-mutated or EBV-associated GCs, possibly exhibiting a synergistic effect when combined with PI3K/AKT inhibitors.

In our study, *Arid1a* deletion induces WNT pathway inactivation in stem/progenitor cells, resulting in abnormal differentiation into metaplastic cells. Regarding the role of *Arid1a* in intestinal stem cells, a previous study demonstrated that *Arid1a* mutation causes WNT pathway inactivation, with *Sox9* downregulation disrupting normal differentiation.^53^ Interestingly, our *Arid1a*-mutant mice exhibited upregulation of *Sox9*, aligning with findings from a previous study involving stomach-specific *Arid1a-*mutant mice.^54^ This discrepancy may reflect SOX9’s function as a principal metaplastic marker in the stomach,^55^ suggesting it may have distinct roles in gastric versus intestinal stem cells. Otherwise, SOX9-expressing metaplastic cells dramatically expand within the type 2 immunity-dominant microenvironment. This hypothesis is further supported by the lack of SOX9 upregulation in APP (Cre) organoids, suggesting that SOX9 activation is modulated by the surrounding microenvironment. Furthermore, prior studies have shown that *Arid1a* heterozygosity facilitates tumor progression through a global loss of enhancers, leading to suppression of the *p53* and apoptosis pathways.^56^ Nonetheless, our novel findings underscore a significant relationship between Arid1a and IL-33, particularly relevant to the type 2 immunity-dominant tumor microenvironment.

Recent studies involving human subjects have indicated that mutations in *ARID1A* exhibit a low correlation with *ARID1A* mRNA and protein expression. In approximately 30%–50% of *ARID1A*-mutated cancers, intact ARID1A protein expression has been observed, although its clinical significance remains underexplored. Moreover, protein expression is more closely associated with patient survival than the mutation itself, as it is linked to biallelic inactivation.^57^^,^^58^ Consequently, our mouse models represent human ARID1A-deficient GCs, rather than all *ARID1A*-mutated cases. Therefore, further research is required to elucidate the impact of *ARID1A* mutation in the absence of functional deletion on tumorigenesis in the future.

*ARID1A* mutations are characterized by an increase in the density of tumor-infiltrating lymphocytes, prompting growing interest in studying type 1 immune responses associated with these mutations for immunotherapy. Consistent with previous studies, *Arid1a*-mutated mice also suggest the activation of type 1 immunity, as indicated by the GSEA and CEBERSORTx results. Therefore, further research is needed to elucidate the relationship between type 1 and type 2 immunity and their impact on tumorigenesis in future studies.

In summary, alterations in *ARID1A* and *PIK3CA* drive type 2 immunity during gastric tumorigenesis, and the activated IL-33/IL-13 axis is a promising therapeutic target.

### Limitations of the study

This study has several limitations. First, experiments using human samples were limited. Future studies employing patient-derived organoids or xenograft models are warranted. Second, the mechanism by which *ARID1A* mutation leads to AKT phosphorylation remains unclear. Further investigations using precise techniques such as assay for transposase-accessible chromatin using sequencing (ATAC-seq) or chromatin immunoprecipitation sequencing are needed, as previous studies have suggested that ARID1A deletion induces epigenetic alterations. Finally, our treatment experiments were mainly conducted on early-stage GCs, so it remains uncertain whether these therapeutic strategies are effective against advanced-stage GCs. Additional research on drug delivery systems and drug selection is required for future clinical applications.

## Resource availability

### Lead contact

Further information and requests for resources and reagents should be directed to and will be fulfilled by the lead contact, Yoku Hayakawa (yhayakawa-tky@umin.ac.jp).

### Materials availability

All the unique reagents generated in this study will be available from the lead contact with a completed material transfer agreement.

### Data and code availability

• Bulk- and single-cell RNA sequence data have been deposited at Zenodo (DOIs: 10.5281/zenodo.15439637, 10.5281/zenodo.15453784, and 10.5281/zenodo.15440585) and are publicly available as of the date of publication.
• This paper does not report original code.
• Further information and requests for resources and reagents should be directed to and will be fulfilled by the lead contact, Yoku Hayakawa (yhayakawa-tky@umin.ac.jp).

## Acknowledgments

We thank Yang Ke for providing us the cell line, GES1. We also thank the staff members at the Laboratory of Gastroenterology, Graduate School of Medicine, University of Tokyo and the Institute of Medical Science, Asahi Life Foundation, especially Sayaka Ito, Hiromi Kato, and Akiko Kikuchi, for their assistance.

This study was supported by Daiwa securities Foundation, Kato Memorial Bioscience Foundation, UBE Foundation, Mochida Memorial Foundation for Medical and Pharmaceutical Research, Takeda Science Foundation, Kobayashi Foundation for Cancer Research, International Medical Research Foundation, and Cell Science Research Foundation (to J.A.); Yakult Bio-Science Foundation; Yasuda Memorial Foundation; Ono Pharmaceutical Foundation for Oncology, Immunology, and Neurology; Eli Lilly Japan KK Innovation Research Grant 2023; Research Grant of the Princess Takamatsu Cancer Research Fund; Suzuken Memorial Foundation, The Mitsubishi Foundation, The Kurata Grants by The Hitachi Global Foundation (to Y. Hayakawa); JSPS KAKENHI Grants-in-Aid for Scientific Research (no. 24K18531 [J.A.], 23H02744 and 24K22113 [Y. Hayakawa]), P-PROMOTE from AMED (JP24ama221332, Y. Hayakawa); the Advanced Research and Development Programs for Medical Innovation (PRIME) (JP24gm6510018, Y. Hayakawa.); Moonshot R & D from JST (no. JPMJMS2214-10 to M.F.); and NIH/NCI funding including R01DK48077 and R35CA210088 (T.C.W.).

## Author contributions

J.A., conceptualization: supporting, data curation: lead, formal analysis: lead, investigation: lead, methodology: lead, validation: lead, visualization: lead, writing – original draft: lead. Y. Hayakawa, conceptualization: lead, data curation: lead, formal analysis: lead, funding acquisition: lead, investigation: lead, methodology: lead, supervision: lead, validation: supporting, visualization: supporting, writing – original draft: lead, writing – review and editing: lead. N.S., formal analysis: supporting, writing – review and editing, supporting information. H.K., formal analysis: supporting, writing – review and editing: supporting information. M.H., data curation: supporting, resources: supporting, writing – review and editing: supporting. Y.M., data curation, resources, writing – review and editing. H.K., data curation, resources, writing – review and editing. S.A., writing – review and editing: supporting information. K.K., writing – review and editing: supporting information. Y.O., writing – review and editing: supporting information. M.T., writing – review and editing: supporting information. S.I., writing – review and editing: supporting information. K.M., formal analysis: supporting, writing – review and editing: supporting information. Y.I., writing – review and editing: supporting information. T.S., writing – review and editing: supporting information. C.S., writing – review and editing: supporting information. C.U., writing – review and editing: supporting information. K.Y., writing – review and editing: supporting information. H.F., writing – review and editing: support. S.K., writing – review and editing: supporting information. H.N., writing – review and editing: supporting information. T.I., writing – review and editing: supporting information. H.T., writing – review and editing: supporting information. T.U., writing – review and editing: supporting information. H.I., writing – review and editing: supporting information. Y. Hirata, writing – review and editing: supporting information. M.K., writing – review and editing: supporting information). G.H.S., resourcing: supporting, writing – review and editing: supporting information. T.C.W., conceptualization: support, supervision: support, writing – original draft: support, writing – review and editing: support. M.F., project administration: support, supervision: support, writing – review and editing: support.

## Declaration of interests

This work was prepared while G.H.S. was employed at Columbia University Irving Medical Center. The opinions expressed in this article are the author’s own and do not reflect the view of the National Institutes of Health, the Department of Health and Human Services, or the United States government.

## STAR★Methods

### Key resources table

**Table:** 

REAGENT or RESOURCE	SOURCE	IDENTIFIER
**Antibodies**		

Rabbit monoclonal anti-*p*-AKT (Ser 473)	cell signaling technology	Cat# 4060; RRID: AB_2315049
Rabbit monoclonal anti-*p*-AKT (Thr 308)	cell signaling technology	Cat# 13038; RRID: AB_2629447
Rabbit polyclonal anti-total-AKT	cell signaling technology	Cat# 9272; RRID: AB_329827
Goat polyclonal anti-IL33	BD biosciences	Cat# AF3626; RRID: AB_884269
Rabbit monoclonal anti-ARID1A	cell signaling technology	Cat# 12354; RRID: AB_2637010
Rabbit monoclonal anti-ICOS	cell signaling technology	Cat# 67223; RRID: AB_3096171
Rabbit monoclonal anti-ST2	Abcam	Ab194113
Rat monoclonal anti-IL13	Invitrogen	2538070
Rabbit monoclonal anti-CD206	cell signaling technology	Cat# 24595; RRID: AB_2892682
Rabbit monoclonal anti-Tryptase	Abcam	ab2378; RRID: AB_303023
Rabbit monoclonal anti-β-actin (HRP conjugated)	cell signaling technology	Cat# 5125; RRID: AB_1903890
Rabbit polyclonal anti-DCLK1	Abcam	Cat# ab31704; RRID: AB_873537
Mouse monoclonal anti-H/K-ATPase	Santa cruz biotechnology	Cat# sc-374094; RRID: AB_10917224
Rabbit monoclonal anti-GIF	Columbia university	NA
Rabbit monoclonal anti-Ki67	cell signaling technology	Cat# 12202; RRID: AB_2620142
Rat monoclonal anti-Ki67	Biolegend	Cat# 151202; RRID: AB_2566621
Rabbit monoclonal anti-CD45	cell signaling technology	Cat# 70257; RRID: AB_2799780
Rabbit monoclonal anti-F4/80	cell signaling technology	Cat# 70076; RRID: AB_2799771
Goat monoclonal anti-CD44v6	Bio RAD	Cat# MCA1967; RRID: AB_323213
Rabbit monoclonal anti-β-catenin	Cell signaling technology	Cat# 9562; RRID: AB_331149
FITC-conjugated Griffonia(Bandeiraea)simplicifolia Lectin 2	VECTOR LABORATORIES	Cat# FL-1211; RRID: AB_2336495
Rabbit monoclonal anti-αSMA	Abcam	Cat# ab5694; RRID: AB_2223021
Rabbit polyclonal anti-RFP	Rockland	Cat# 600-401-379; RRID: AB_2209751
Goat polyclonal anti-RFP	Rockland	Cat# 200-101-379; RRID: AB_2744552
Goat polyclonal anti-GFP	Abcam	Cat# ab5450; RRID: AB_304897
Goat polyclonal anti-CLUSTERIN	RD bioscience	Cat# AF2747; RRID: AB_2083314
Mouse monoclonal anti-MUC5AC	Abcam	Cat# ab3649; RRID: AB_2146844
Rabbit monoclonal anti Axin2	Abcam	Cat# ab109307; RRID: AB_10862550
Rabbit polyclonal anti-TFF2	sino biological	200208-T08

**Bacterial and virus strains**		

pAV[shRNA]-EGFP-U6>mArid1a[shRNA#1]	Vectorbuilder	VB900160-4526tfr
pAV[shRNA]-EGFP-U6>hARID1A[shRNA#1]	Vectorbuilder	VB900140-0426khw
pLV[Exp]-EGFP:T2A:Puro-EF1A > mCRE	Tokyo University	NA

**Biological samples**		

Human Stomach adenocarcinoma tissue array	US Biomax, inc.	BS01011b

**Chemicals, peptides, and recombinant proteins**		

Tamoxifen	Sigma-Aldrich	T5648-1G
PS-48	Selleck	S7586
MK-2206	Selleck	S1075
Recombinant mouse IL33	Biolegend	580502
Anti-IL33 antibody	BD biosciences	Cat# AF3626, RRID:AB_884269
Anti-IL13 antibody	Invitrogen	Cat# 16-7135-81, RRID:AB_763562
Anti-ICOS antibody	Bio X Cel	Cat# BE0059, RRID:AB_1107622

**Critical commercial assays**		

RNAscope(R) 2.5 HD Duplex Detection Reagents Kit	ACD	ADC 322430
NucleoSpin RNA	MACHEREY-NAGEL	740955.10
ISOGEN with a Spin Column kit	Nippon Gene	318–07511
VECTASTAIN Elite ABC Kit	VECTOR LABORATORIES	PK-6100
Cell-counting kit 8	Dojindo Laboratories	341–07761

**Deposited data**		

Raw and analyzed data (Bulk RNA seq on mice models)	This paper	https://doi.org/10.5281/zenodo.15439637
Raw and analyzed data (Bulk RNA seq on organoids)	This paper	https://doi.org/10.5281/zenodo.15453784
Raw and analyzed data (Single cell RNA seq on mice models)	This paper	https://doi.org/10.5281/zenodo.15440585

**Experimental models: Cell lines**		

Human: AGS	ATCC	N/A
Human: GES1	Beyotime Biotechnology	N/A

**Experimental models: Organisms/strains**		

C57BL/6 (Arid1a^flox/flox^)	Jackson lab	
C57BL/6 (Cdkn2a^flox/flox^)	Columbia university	
C57BL/6 (TFF1-cre-Tg)	Tokyo university	
C57BL/6(PTEN^flox/flox^)	Jackson lab	
129/C57BL/6(LSL-KrasG12D/+)	Jackson lab	
C57BL/6(CDH1^flox/flox^)	Jackson lab	
B6.129-Lgr5tm1(cre/ERT2)Cre	Jackson lab	
B6; 129S6-Gt(ROSA)26Sortm14(CAG-tdTomato)Hze/J	Jackson lab	
C57BL/6 (Loxp-STOP-Loxp (LSL)-Pik3ca^H1047R)^	Jackson lab	

**Oligonucleotides**		

Primer: Arid1a Forward: 5′-CTG TTG CCA TGC ATG TTG CT-3′	This paper	N/A
Primer: Arid1a Reverse: 5′-CCC ATC ATG CCC CCT TGA TT-3′	This paper	N/A
Primer: Cdkn2a Forward: 5′-CGC AGG TTC TTG GTC ACT GT-3′	This paper	N/A
Primer: Cdkn2a Reverse: 5′-TGT TCA CGA AAG CCA GAG CG-3′	This paper	N/A
Primer: GAPDH Forward: 5′-AGG TCG GTG TGA ACG GAT TTG-3′	This paper	N/A
Primer: GAPDH Reverse: 5′-GGG GTC GTT GAT GGC AAC A-3′	This paper	N/A

**Software and algorithms**		

GraphPad Prism version 8.0.0 for Windows	GraphPad	https://www.graphpad.com/guides/prism/latest/user-guide/citing_graphpad_prism.htm
CLC Genomics Workbench software v7.5	Qiagen	https://digitalinsights.qiagen.com/products-overview/discovery-insights-portfolio/analysis-and-visualization/qiagen-clc-genomics-workbench/
R studio	RStudio, PBC	https://posit.co/download/rstudio-desktop/

### Experimental model and study participants details

#### Mice

*Arid1a*^*flox/flox*^, *Loxp-STOP-Loxp* (LSL)-*Pik3ca*^*H1047R*^, *Pten*^*flox/flox*^, LSL-*Kras*^*G12D*^*, Cdh1*^*flox/flox*^, *Rosa26*-LSL-*tdTomato*, and *Lgr5*-creERT mice were purchased from Jackson Laboratories (Bar Harbor, ME, USA). *Cdkn2a (p16)*^*flox/flox*^ mice^59^ and *Tff1-*cre^32^ mice have been described previously. All analyses were conducted using male 3-month-old mice, except where otherwise indicated in the figure legends. Male mice were exclusively used in this study to avoid the potential confounding effects of hormonal fluctuations associated with the estrous cycle in females. As a result, sex-based differences could not be evaluated, which is a limitation of the study. Mice were maintained in a specific-pathogen-free environment in the Animal Care Facility of the University of Tokyo. All animal studies and procedures were approved by the ethics committee of the University of Tokyo (ID: 24-2).

#### Organoid generation

Organoids were generated as described previously.^24^^,^^60^ The stomachs were removed from male mice, cut longitudinally, washed with cold PBS, and incubated in PBS containing 8 mM EDTA for 60 min on ice. Gland fractions were mechanically dissociated by coverslip, centrifuged at 900 rpm for 6 min at 4°C, and diluted with advanced DMEM/F12 (Invitrogen, Waltham, MA, USA) containing B27, N2, 1 μM n-Acetylcysteine, 10 mM HEPES, penicillin/streptomycin, and Glutamax (all from Invitrogen). The glands were embedded in Matrigel and 500 glands/well were seeded in a pre-warmed 24-well plate. After Matrigel solidified, it was overlaid with advanced DMEM/F12 containing 50 ng/mL EGF (Invitrogen) and conditioned medium containing noggin, R-spondin, and Wnt3a. The medium was replaced twice weekly. Growth factors were removed when the proliferation was analyzed. Specifically, we generated *Arid1a*^*flox/flox*^, *Cdkn2a (p16)*^*flox/flox*^, and LSL-*Pik3ca*^*H1047R*^ organoids from mice harboring the same gene mutations. These organoids were then infected with a lentivirus expressing Cre, leading to the development of mutated organoids (hereafter referred to as APP (Cre) organoids).

#### Cell lines

Human GC cell lines (AGS and GES1) were cultured in Ham’s F-12 or RPMI medium supplemented with 10–20% FBS. Cells were maintained as a monolayer at 37°C in a humidified atmosphere containing 5% CO_2_. Cell numbers were determined using a Cell Counting Kit-8 (Dojindo Laboratories, Kumamoto, Japan) according to the manufacturer’s protocol. AGS was obtained from female patients, whereas the host gender of GES1 was unspecified.^61^ The cell lines have not been authenticated since purchase; however, they were tested and confirmed to be negative for mycoplasma contamination.

### Method details

#### Treatment *in vivo*

*Helicobacter pylori (H. pylori;* PMSS1) was orally administered to induce *H. pylori* infection.^62^^,^^63^^,^^64^^,^^65^ To neutralize IL33, the antibody against IL33 was administered intraperitoneally at a dose of 3.6 μg/body 3 times weekly for 4 weeks. To deplete ICOS-positive ILC2, the antibody against ICOS was administered intraperitoneally at a dose of 100 μg/body twice weekly for 4 weeks. To neutralize IL13, the antibody against IL13 was administered intraperitoneally at a dose of 20 μg/body 3 times weekly for 4 weeks. Lineage-tracing experiments were performed as reported previously.^66^

#### Treatment *in vitro*

An AKT inhibitor (MK-2206: 150 nM) and a PDK1 agonist (PS48: 10 μM) was directly added to the medium of cell lines. After 24 and 48 h, viable cell numbers were analyzed using the Cell Counting Kit-8 (Dojindo Laboratories, Mashiki, Japan) according to the manufacturer’s protocol. For organoid experiments, the AKT inhibitor (MK-2206) was also added directly to the organoid medium (150 nM). Organoid diameters were analyzed after 6 and 11 days. To elucidate the effect of IL33 *in vitro*, recombinant IL33 was added to the organoid medium (10 mg/mL). Organoid diameters were analyzed after 6 and 11 days.

#### Mouse xenograft tumor model

Xenograft mouse model experiments were performed as previously described.^67^ APP (Cre) organoid cells (1 × 10^6^) were resuspended in 50% Matrigel/PBS (200 μL) and injected subcutaneously into nude mice. The animals were sacrificed on day 29 and the tumors were collected and weighed.

#### Adenovirus infection

Cells were seeded into 12-well plates and after 24 h, infected with adenovirus (arranged for MOI = 100) and directly added to the medium. After 24 h, the medium was replaced with a medium without FBS. After 24 h, cells without the selection procedure were harvested or analyzed using a cell proliferation assay.

For organoids, adenoviruses (arranged for MOI = 100) were suspended in organoid cells after dissolution in Matrigel. After 3 h, organoids were seeded in 24-well plates. After 48 h, the medium was replaced with medium without growth factors. Organoids without the selection procedure were harvested after 24 h and their diameters were calculated for proliferation analysis after 6 and 11 days.

The schema of adenovirus vectors is shown in Figure S15.

The gene expression for both cell lines and organoids was analyzed by western blotting.

#### Lentivirus infection

Organoids were dissociated from Matrigel using TrypLE (Thermo Fisher Scientific, USA) to generate a single-cell suspension. The dissociated cells were then resuspended in a solution containing pLV-EF1-Cre-2a-vsfGFP-2a-puro lentivirus at an optimized concentration for infection. Following this, the cell suspension was centrifuged at 200g for 30 min to facilitate viral contact and enhance infection efficiency. After centrifugation, the cells were re-embedded in Matrigel and cultured under standard conditions. After 48 h, the medium was replaced with puromycin to select infected cells. After 24 h, cells were passaged repeatedly. This procedure allowed stable lentiviral integration, enabling subsequent analysis and selection of infected cells.

#### Western blotting

Protein lysates were prepared from tissues or cultured cells, separated using sodium dodecyl sulfate-polyacrylamide gel electrophoresis, and transferred onto polyvinylidene difluoride membranes (Merck Millipore, Burlington, MA, USA). Membranes were probed with primary antibodies and incubated with secondary antibodies. Immunocomplexes were detected using an enhanced chemiluminescence system (Amersham Biosciences, Amersham, UK). The primary antibodies used were IL33 (RD Bioscience, San Diego, CA, USA; 1:1000), *p*-AKT (Ser473) (Cell Signaling Technology, Danvers, MA, USA; 1:1000), *p*-AKT (Thr308) (Cell Signaling Technology; 1:1000), Total-AKT (Cell Signaling Technology; 1:1000), ARID1A (Cell Signaling Technology; 1:1000), CTNNB1 (Cell Signaling Technology; 1:1000), AXIN2 (Abcam; 1:1000) and β-actin (Sigma Aldrich, St. Louis, MO, USA; 1:10000).

#### Histopathologic analysis

Gastric tissues from mice were fixed in either 10% formalin or 4% paraformaldehyde, embedded in paraffin or optimum cutting temperature compound, respectively, and cut into 5-μm sections. Immunohistochemistry was performed as previously described.^19^ *In situ* hybridization was performed as described previously^68^ or using an RNAscope kit (Advanced Cell Diagnostics, Newark, CA, USA) with *Lgr4* and *Lgr5* probes. The average number of positive cells across 20 glands per mouse at the indicated sites was quantified. Immunostained samples were imaged using Leica DMi8 (Leica Microsystems), VS200 (Evident), and BZ-X800 (Keyence) fluorescence microscopy systems.

#### Antibodies for immunohistochemistry

The following primary antibodies were used: ARID1A (Cell Signaling Technology; 1:200), Ki67 (Cell Signaling Technology; 1:200), CD45 (Cell Signaling Technology; 1:200), IL33 (RD Bioscience; 1:200), MUC5AC (Abcam; 1:200), ICOS (Cell Signaling Technology; 1:200), ST2 (Abcam; 1:200), IL13 (Abcam; 1:200), CD206 (Cell Signaling Technology; 1:200), Tryptase (Abcam; 1:200), DCLK1 (Abcam; 1:200), β-catenin (Cell Signaling Technology; 1:200), F4/80 (Cell Signaling Technology; 1:200), *p*-AKT at Ser473 (Cell Signaling Technology; 1:200), H/K-ATPase (Santa Cruz Biotechnology, Santa Cruz, CA, USA; 1:200), GIF (Columbia University, New York, NY, USA; 1:200), CD44v6 (Bio-RAD, Hercules, CA, USA; 1:200), GSII (Vector Laboratories, Newark, CA, USA; 1:200), Clusterin (RD bioscience; 1:200), TFF2 (Sino Biological, Beijing, China; 1:200), RFP (Rockland; 1:200), GFP (Abcam; 1:200) and αSMA (Cell signaling Technology; 1:200).

#### RNA sequence

RNA was extracted from the gastric mucosa of mice using a NucleoSpin RNA kit (MACHEREY-NAGEL GmbH & Co., Duren, Germany) or cultured using ISOGEN with a Spin Column kit (Nippon Gene, Tokyo, Japan). RNA was sequenced using the NovaSeq 6000 system. Data were analyzed on a log2 scale using the Subio platform (Kagoshima, Japan) or RaNA-seq website.^69^ The significance of the differential expression was analyzed using the Wald test (DESeq2). Differentially expressed transcripts were defined as those with a Wald test *p*-value <0.05, >2-fold upregulation, or <0.5-fold downregulation. GSEA was performed as described previously.^70^ The significance (*p* value) and false discovery rate (Q value) of the enrichment scores were determined using 1000 permutations of random gene sets of comparable sizes.

#### Quantitative PCR

Total RNA was extracted from whole stomach samples using a NucleoSpin RNA kit (MACHEREY-NAGEL) and subjected to first-strand complementary DNA synthesis using the Superscript III cDNA Amplification System (Invitrogen, Waltham, MA, USA). RT-qPCR was performed on an ABI 7300 system using SYBR Green (Roche, Basel, Switzerland). The results are expressed as the copy number of each gene relative to that of *Gapdh*.

#### Human tissue array

IL33 and ARID1A protein expression in human GC tissues was quantified by the proportion of positive cells and staining intensity as described previously.^60^ Briefly, IL33-positive cells in cancer cells were scored as follows: 1, <5% positive cells; 2, 5–50% positive cells; 3, >50% positive cells. Two experts independently evaluated the immunohistochemical labeling in each case, and there was no disagreement.

#### Fluorescence-assisted cell sorting

The gastric mucosa was isolated from the mice and dissociated into single cells by stirring in HBSS containing a mixture of collagenase and dispase. After that, the isolated single cells were incubated with the specific antibodies. Finally, Mouse ILC2s stained with CD45/Lineage/CD90.2/ICOS antibodies were sorted from WT and APP mice using FACS, as described previously.^60^

#### Analysis of the Cancer Genome Atlas dataset

TCGA dataset was analyzed using Phantasus v1.19.3 (https://ctlab.itmo.ru/phantasus/).^71^ GSEA was performed as described previously.^70^ The significance (*p* value) and false discovery rate (Q value) of the enrichment scores were determined using 1000 permutations of random gene sets of comparable sizes.

#### The analysis of single-cell RNA-seq

Droplet-based single-cell partitioning and single-cell RNA-Seq libraries were generated using Chromium Fixed RNA Profiling Reagent Kits (10× Genomics, Pleasanton, CA, USA) according to the manufacturer’s protocol, based on 10× GemCode proprietary technology.

Briefly, fresh gastric antrum specimens from WT and Arid mice were extracted and immediately frozen in liquid nitrogen. After fixing at least 25 mg of frozen tissue with 4% formaldehyde solution, cells were separated from the tissue using a gentleMACS Octo Dissociator (Miltenyi Biotec, Bergisch Gladbach, Germany) in a dissociation solution (Gibco RPMI RPMI 1640 + 0.2 mg/mL Liberase; Gibco, Waltham, MA, USA). The dissociated tissue was passed through a 30 μm filter to remove debris and undissociated tissue pieces.

Whole transcriptome probe pairs, consisting of the left-hand side and right-hand side of each targeted gene, were added to the fixed sample. The probe pairs hybridized to their complementary target RNA during overnight incubation.

After hybridization, the samples are washed three times, single-cell suspension at a density of some 3500 cells/μL was mixed with GEM master mix and immediately loaded together with Single Cell Fixed RNA Gel Bead and Partitioning Oil into a Chromium Next GEM Chip Q. The chip was loaded onto Chromium X (10× Genomics) for single-cell GEM generation and barcoding. Once the ligation and barcoding steps were completed, the GEMs were broken down by the addition of a recovery agent, and a PCR master mix was added directly to the post-GEM aqueous phase to pre-amplify the ligated products. The pre-amplified products were cleaned using SPRIselect (Beckman Coulter, Brea, CA, USA).

The 10x barcoded, ligated probe products were indexed using Sample Index PCR. The generated final library molecules were cleaned up by SPRIselect and incorporated into the finished library, which was compatible with Illumina next-generation short-read sequencing. Size profiles of the sequencing library were examined using an Agilent Bioanalyzer 2100 with a high-sensitivity DNA chip (Agilent, Santa Clara, CA, USA).

The libraries were sequenced on an Illumina NovaSeq X Plus system using the NovaSeq X Series 10 B Reagent Kit (Illumina, San Diego, CA, USA) with a paired-end, dual indexing (28/10/10/90-bp) format according to the recommendations of 10× Genomics.

The filtered count matrices were imported into R for further analysis using the Seurat package.^71^ We performed clustering according to the cell markers, generated a heatmap with the top five DEGs, counted cell numbers in each cluster, checked the cell markers with violin plot, bubble plot, and feature plot, and conducted differential expression and GSEA on pseudobulk workflow by using “AggregateExpression'' function in Seurat and R packages DESeq2 and fgsea.

Human single-cell RNA-seq data for GC were obtained from the GSE183904 dataset.^35^ Filtered count matrices were imported into R for further analysis using the Seurat package.^71^ Dimensionality was reduced using PCA and UMAP embedding, with 30 principal components and a resolution of 1.0. The epithelial cells were then divided into subsets based on epithelial markers. ARID1A expression in tumor epithelial cells were obtained using the “AverageExpression” function in Seurat. The patients were then classified as ARID1Ahigh or ARID1Alow. Then we conducted differential expression and GSEA on pseudobulk workflow by using “AggregateExpression'' function in Seurat and R packages DESeq2 and fgsea.

### Quantification and statistical analysis

Continuous variables were expressed as a means with 95% standard errors, whereas categorical variables were expressed as numbers and frequencies (%). The differences between the means were compared using either Student’s t test or the Wilcoxon test. The Kaplan–Meier method was used to calculate the cumulative probability of mortality at 1 year for APP and Apik mice. Statistical significance was set at *p* < 0.05. All of the statistical details of experiments can be found in Figure legend.

## Supplemental information

Document S1. Figures S1–S15

Table S1. Genes with differentially upregulated expression in Arid1a-mutated mice, related to Figure 2

Table S2. Genes with differentially downregulated expression in Arid1a-mutated mice, related to Figure 2

Table S3. Genes with differentially upregulated expression in Arid1a-mutated mice analyzed with a Venn diagram, related to Figure 2

Table S4. Gene set enrichment analysis with Hallmark gene sets between WT and Arid1a-mutated mice at the antrum based on the RNA sequence database, related to Figure 2

Table S5. Gene set enrichment analysis with KEGG gene sets between WT and Arid1a-mutated mice at the antrum based on the RNA sequence database, related to Figure 2

Table S6. CIBERSORTx analysis with RNA sequence analysis, related to Figure 2

Table S7. Gene set enrichment analysis with immunogram gene sets between WT and Arid1a-mutated mice at the antrum based on the RNA sequence database, related to Figure 2

Table S8. Gene set enrichment analysis with Hallmark gene sets between WT and APP (Cre) organoids based on the RNA sequence database, related to Figure 5

Table S9. Genes with differentially upregulated expression in APP (Cre) organoids, related to Figure 5

Table S10. Genes with differentially downregulated expression in APP (Cre) organoids, related to Figure 5

Table S11. Gene set enrichment analysis with immunogram gene sets based on the TCGA database, related to Figure 8

Table S12. Gene set enrichment analysis with Hallmark and KEGG gene sets based on the TCGA database, related to Figure 8
