# Supplementary material for: ARID1A mutation drives gastric tumorigenesis via activating type 2 immune dominant microenvironment
Source: iScience. 2025 Jul 15;28(8):113117. doi: 10.1016/j.isci.2025.113117 (PMC12319253; doi:10.1016/j.isci.2025.113117)
Supplement: Document S1. Figures S1–S15 [file mmc1.pdf]

iScience, Volume *28*

Supplemental information

ARID1A mutation drives gastric tumorigenesis via activating type 2 immune dominant microenvironment

Junya Arai, Yoku Hayakawa, Nobumi Suzuki, Hiroto Kinoshita, Masahiro Hata, Ken Kurokawa, Yuki Matsushita, Sohei Abe, Yukiko Oya, Mayo Tsuboi, Sozaburo Ihara, Yusuke Iwata, Keita Murakami, Toshiro Shiokawa, Chihiro Shiomi, Chie Uekura, Keisuke Yamamoto, Hiroaki Fujiwara, Satoshi Kawamura, Hayato Nakagawa, Tsuneo Ikenoue, Hiroaki Tateno, Tetsuo Ushiku, Hideaki Ijichi, Yoshihiro Hirata, Masato Kasuga, Gloria H. Su, Timothy C. Wang, and Mitsuhiro Fujishiro

Supplementary Figure 1. Histology and immunohistochemical analysis of *Arid1a*-mutated mice, related to Figure 1.

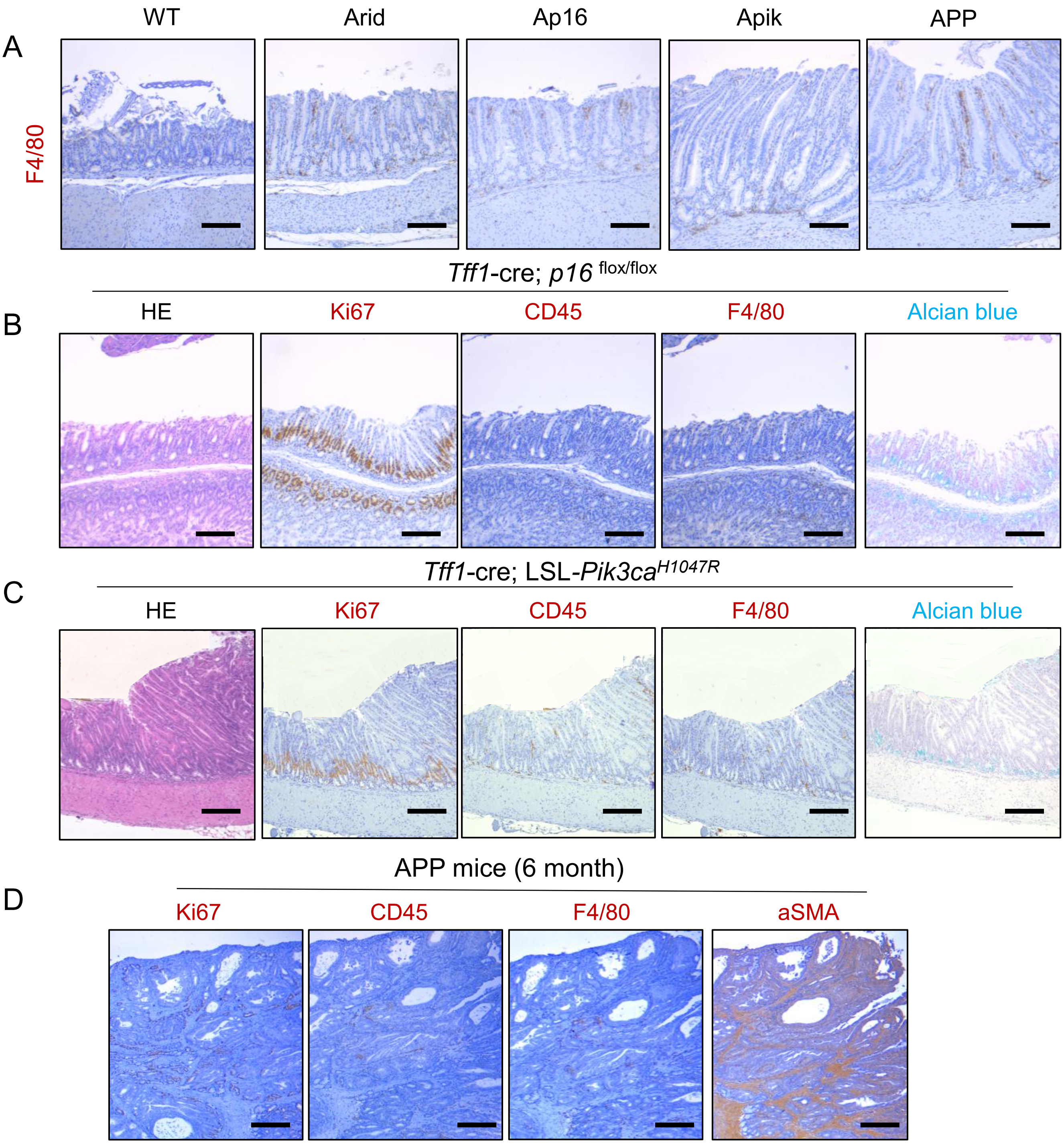

(A) F4/80 staining in WT, Arid, Ap16, Apik, and APP mice. The images are similar but not identical to CD45 staining in the Figure 1E, as they are serial sections prepared from the same paraffin block derived from the same specimen (n = 4/group).

(B) HE, Ki67, CD45, F4/80, and Alcian blue staining in *Tff1*-cre; *p16* $^{flox/flox}$ mice (independent repeats, n = 3).

(C) HE, Ki67, CD45, F4/80, and Alcian blue staining in *Tff1*-cre; LSL-*Pik3ca*^{H1047R} mice (independent repeats, n = 3).

(D) Ki67, CD45, F4/80, and αSMA staining in APP mice aged 6 months (independent repeats, n = 3).

Scale bars represent 100 μm.

Supplementary Figure 2. Histology and immunohistochemical analysis of the gastric corpus of *Arid1a*-mutated mice, related to Figure 1.

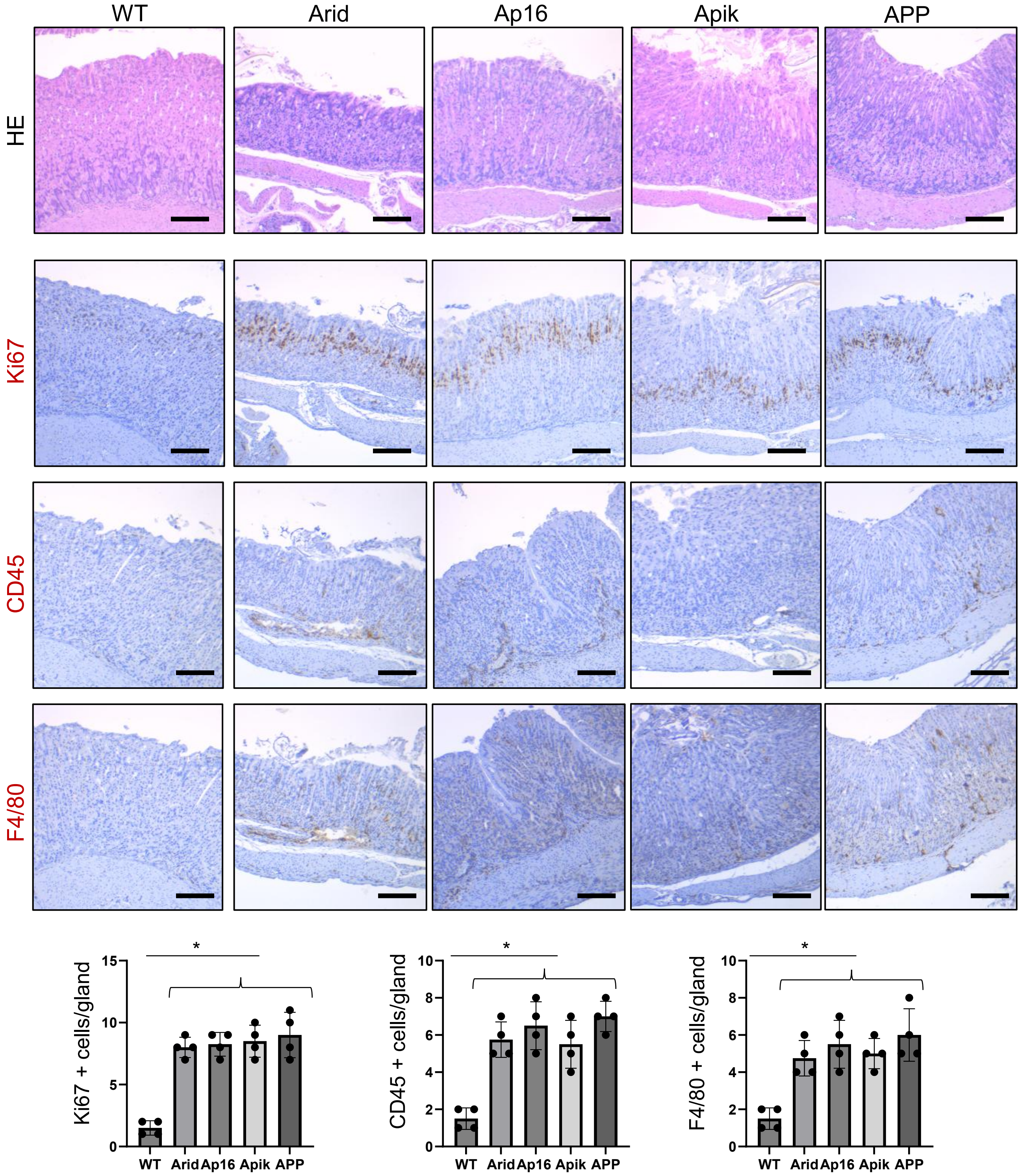

HE, Ki67, CD45, F4/80, and Alcian blue staining of the corpus of WT, Arid, Ap16, Apik, and APP mice.
Numbers of Ki67, CD45, F4/80-positive cells are quantified and shown at the bottom (n=4 mice per group).
The p-value was calculated using a t-test.
Scale bars represent 100 μm. Mean ± S.E.M. *P < .05.

Supplementary Figure 3. Additional analysis with bulk RNA-seq, related to Figure 2.

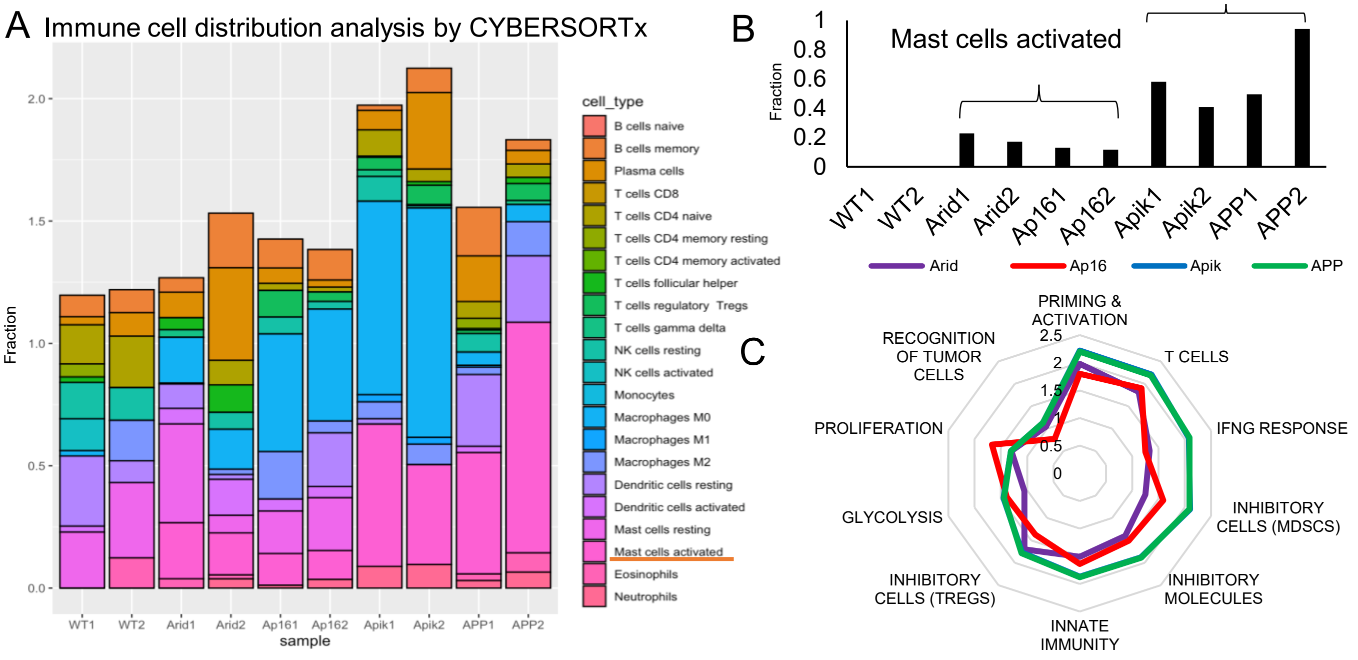

(A-B) Analysis of the inflammatory cells with CIBERSORTx (A) shown as stacked bar graph and (B) the comparison of activated mast cell scores.
(C) GSEA analysis with immunogram gene sets shown as radar chart.

Supplementary Figure 4. Single-cell RNA-seq analysis of the mouse stomach, related to Figure 3.

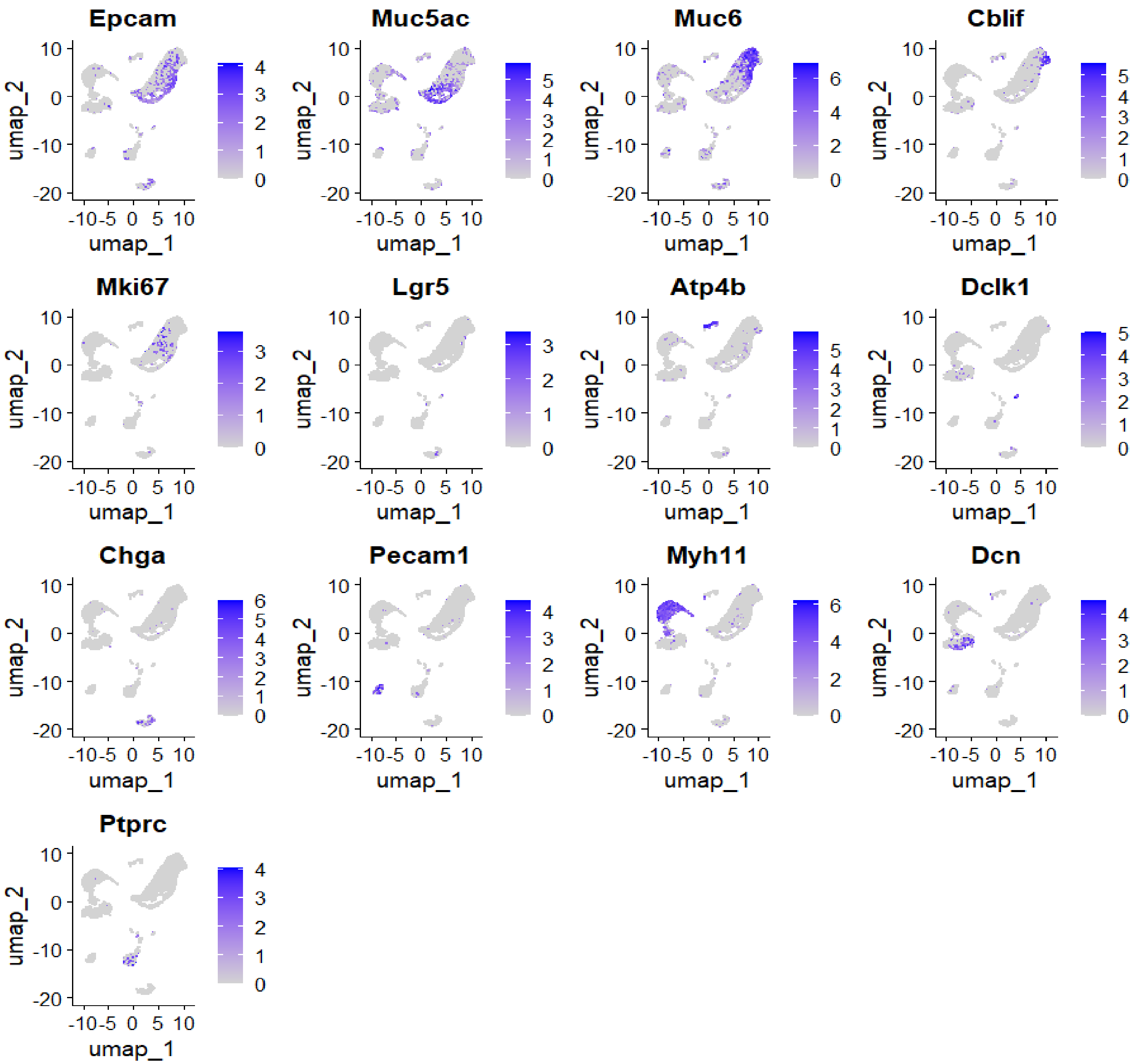

UMAP feature plots colored by the expression of the indicated genes.

Supplementary Figure 5. Single-cell RNA-seq analysis of the pit cell lineage epithelial cells from *Arid1a*-mutated mice and the littermate controls, related to Figure 4.

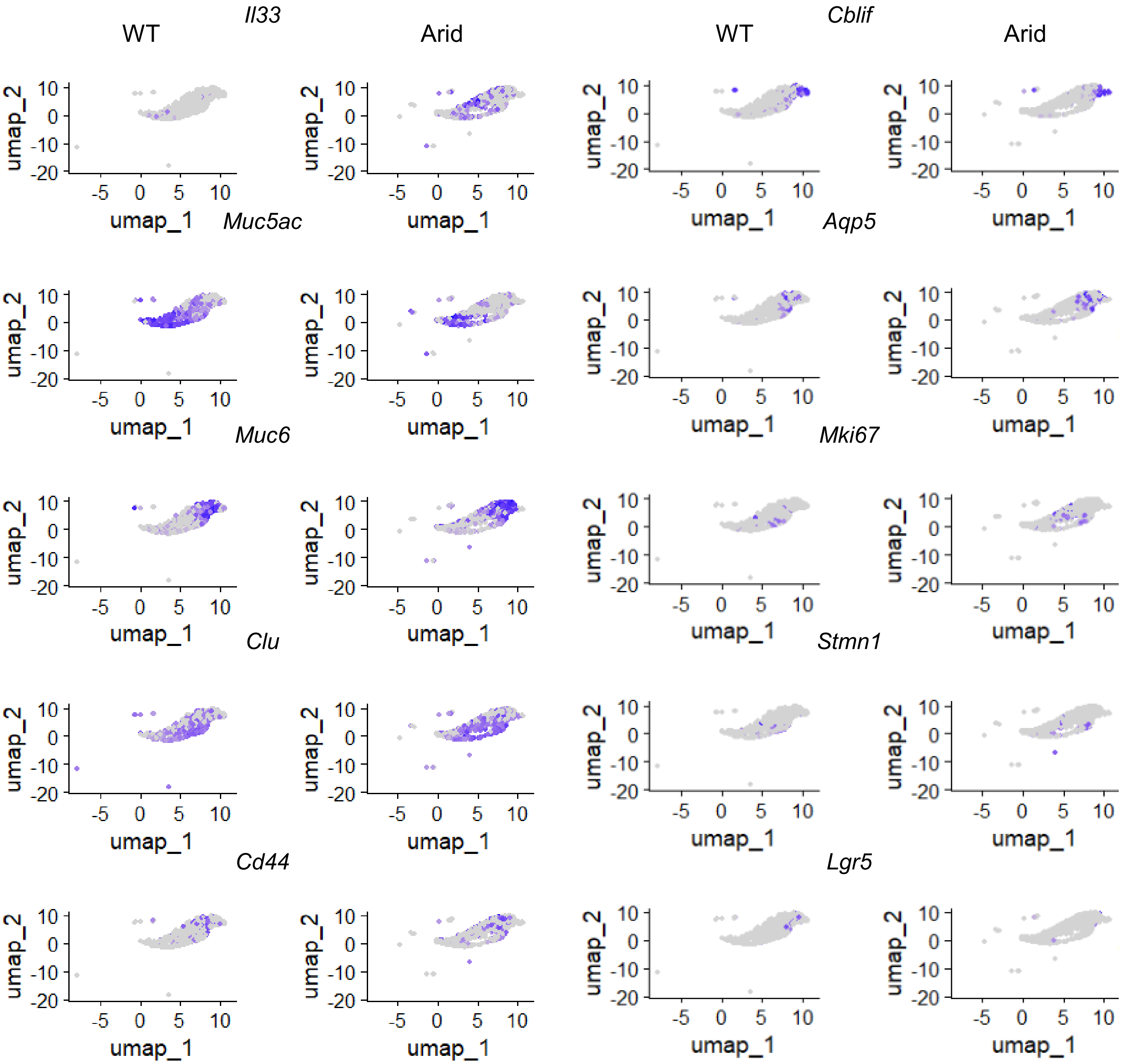

UMAP feature plots colored by the expression of the indicated genes.

Supplementary Figure 6. Metaplastic marker expression in *Arid1a*-mutated mice, related to Figure 4.

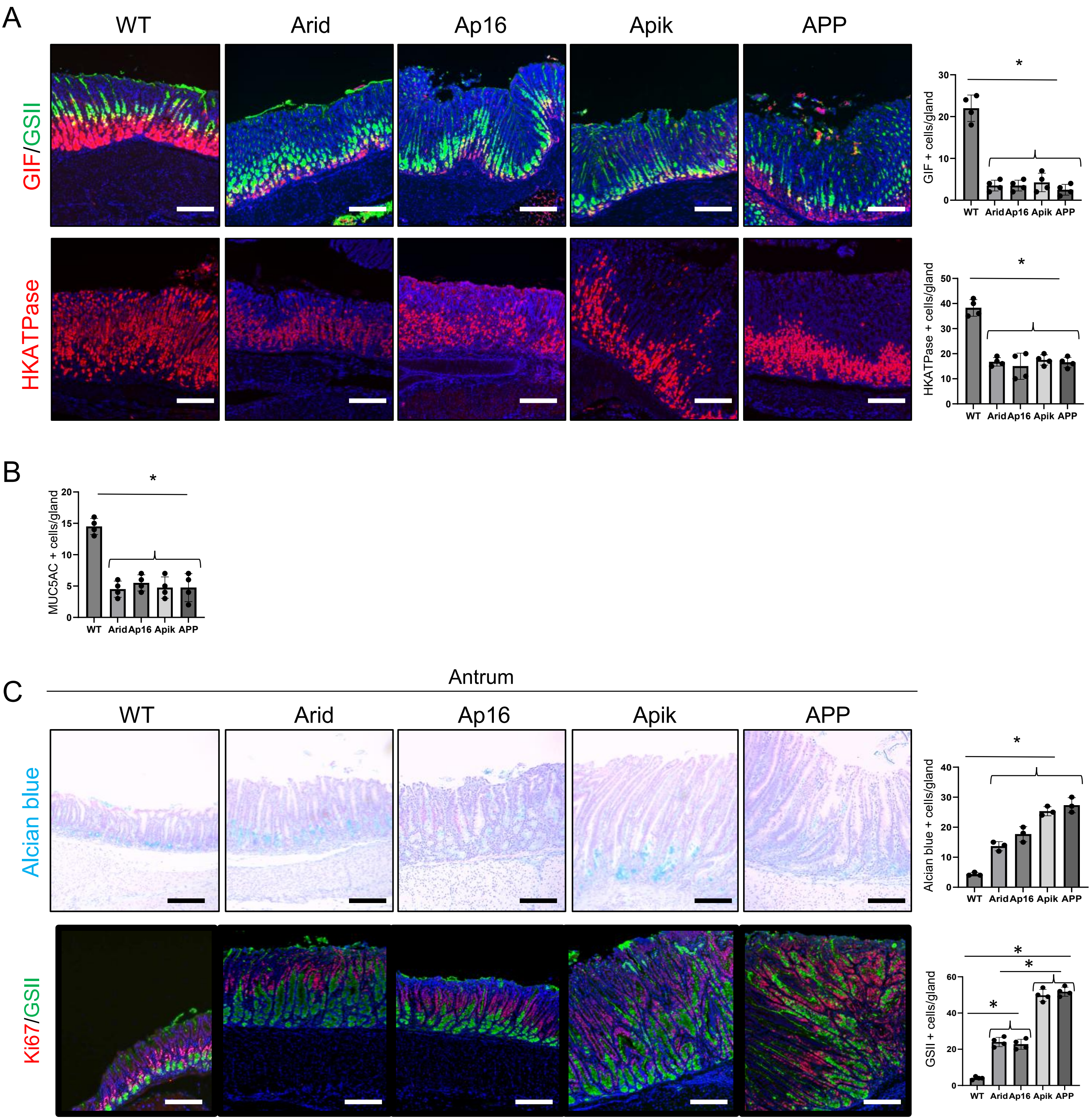

(A) GIF (red)/GSII (green) (top) and HKATPase (red, bottom) staining in WT, Arid, Ap16, Apik, and APP mice. Numbers of GIF and HKATPase positive cells are quantified and shown at the right (n=4 mice per group).

(B) Numbers of MUC5AC-positive cells are quantified (n=4 mice per group).

(C) Alcian blue (light blue, top), and GSII (green)/Ki67 (red) (bottom) staining in WT, Arid, Ap16, Apik, and APP mice. Numbers of Alcian blue and GSII-positive cells are quantified and shown at the right (n=4 mice per group).

The p-value was calculated using a t-test.

Scale bars represent 100 µm. Mean ± S.E.M. *P < .05.

Supplementary Figure 7. Generation of APP (Cre) organoids, related to Figure 5.

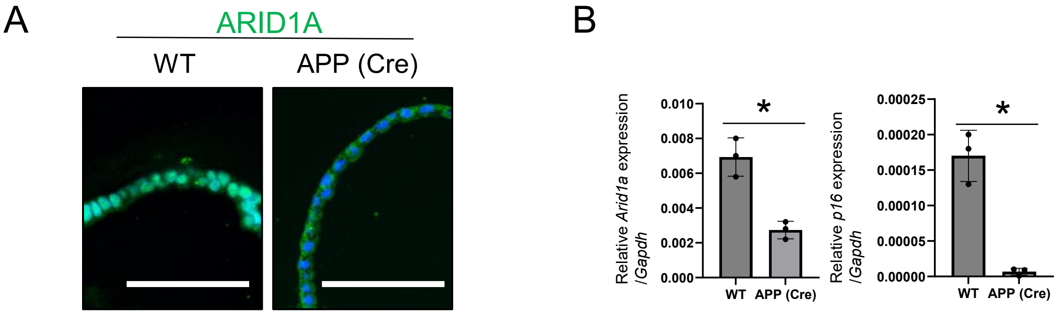

(A) ARID1A staining in WT and APP (Cre) organoids (n = 20/group).
(B) Quantitative PCR analysis of *Arid1a* and *p16* primers in the WT and APP (Cre) organoids. Relative expression per *Gapdh* was calculated (n = 3 per group).

The p-value was calculated using a t-test.
Scale bars represent 100 μm. Mean ± S.E.M. *P < .05.

Supplementary Figure 8. Infiltration of epithelial and inflammatory cells involved in the type II immunity, related to Figure 6.

A

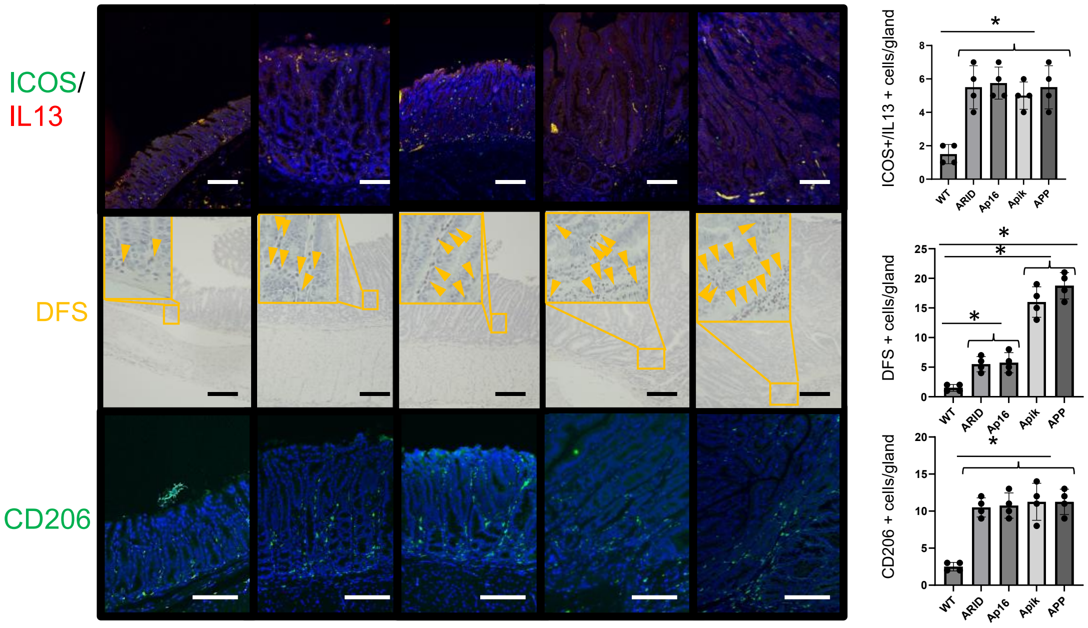

B

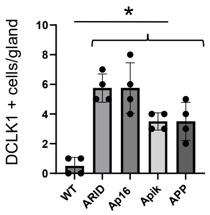

(A) ICOS (green)/IL13 (red), direct fast scarlet (DFS) (brown), and CD206 (green) staining in WT and Arid/Ap16/Apik/APP mice. Numbers of cells expressing each marker are quantified and shown at the right (n=4/group).

(B) Numbers of DCLK1-positive cells are quantified (n=4 mice per group).

The p-value was calculated using a t-test.

Scale bars represent 100 μm. Mean ± S.E.M. *P < .05.

Supplementary Figure 9. *Helicobacter pylori* infection to *Tff1*-cre; *Arid1a*$^{flox/flox}$ mice, related to Figure 6.

A

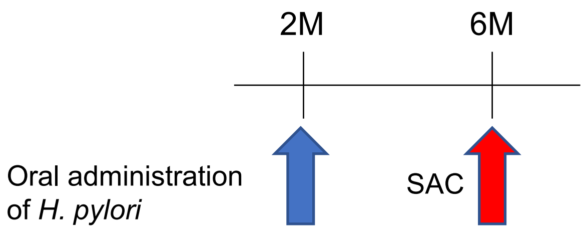

B

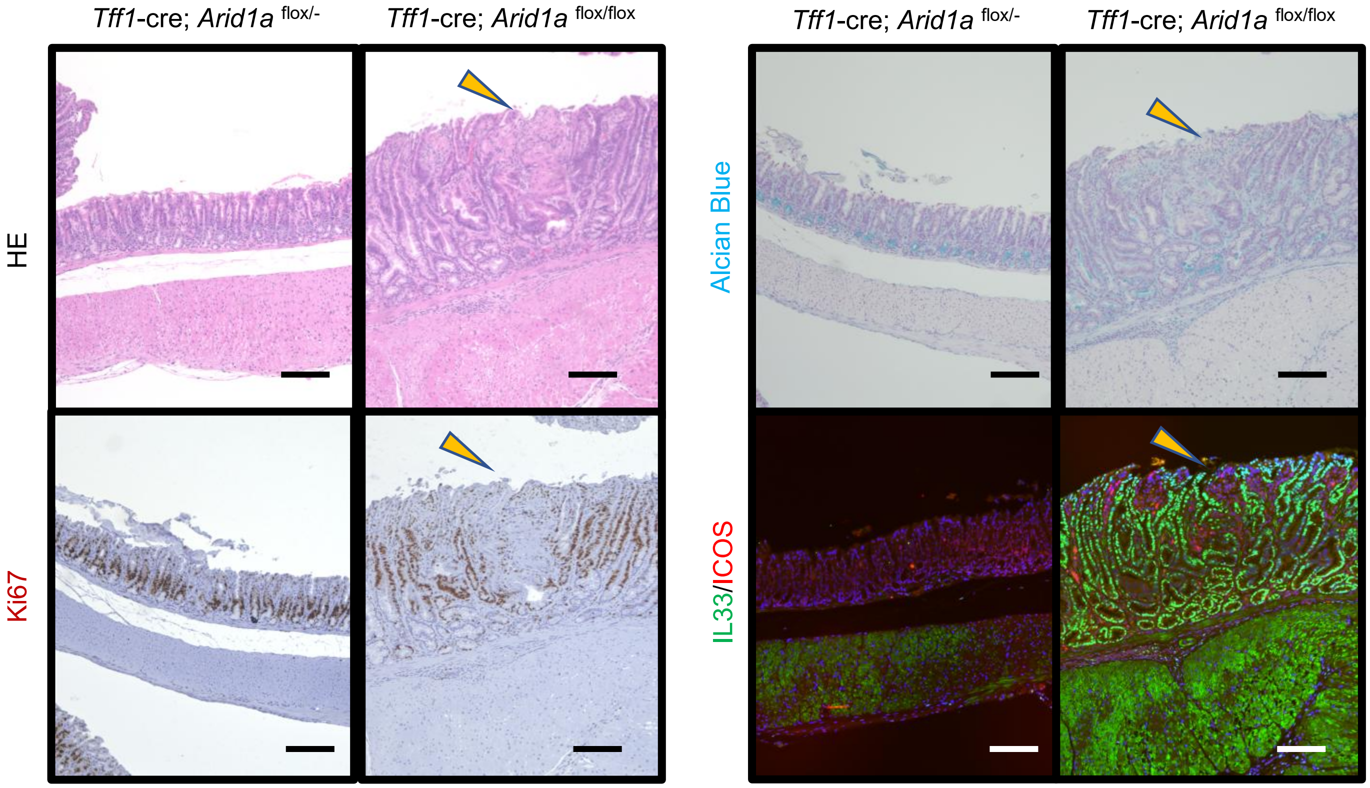

(A)The protocol of the *Helicobacter pylori* (PMSS1) infection.
(B) HE, Ki67 (brown), Alcian blue, and IL33 (green)/ICOS (red) staining in *Tff1*-cre; *Arid1a*$^{flox/flox}$ and *Tff1*-cre; *Arid1a*$^{flox/-}$ mice treated with *Helicobacter pylori* (PMSS1) (independent repeats, n = 3).
Scale bars represent 100 μm. Arrow heads indicate dysplastic lesions.

Supplementary Figure 10. Macroscopic and histological images of *Arid1a/Pten*-mutated mice, related to Figure 6.

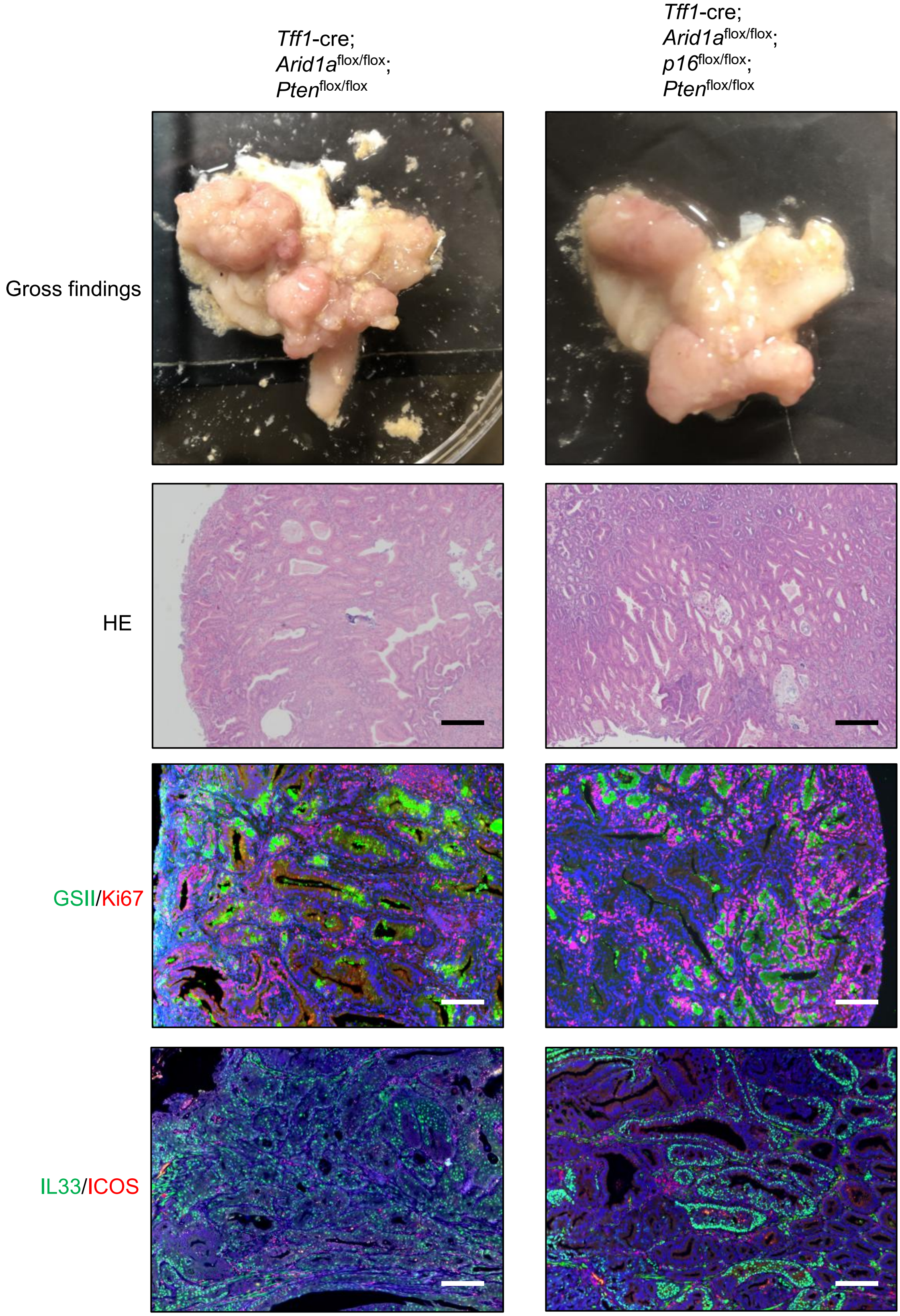

Gross findings and HE, GSII/Ki67, and IL33/ICOS staining of *Tff1*-Cre, *Arid1a*$^{flox/flox}$, *Pten*$^{flox/flox}$ *and Tff1*-Cre, *Arid1a*$^{flox/flox}$; *p16*$^{flox/flox}$, *Pten*$^{flox/flox}$ *mice* (independent repeats, n = 4). Scale bars represent 100 µm.

Supplementary Figure 11. The effects by *Arid1a* mutation on *Kras*-mutated and *Cdh1*-mutated mouse stomach, related to Figure 6

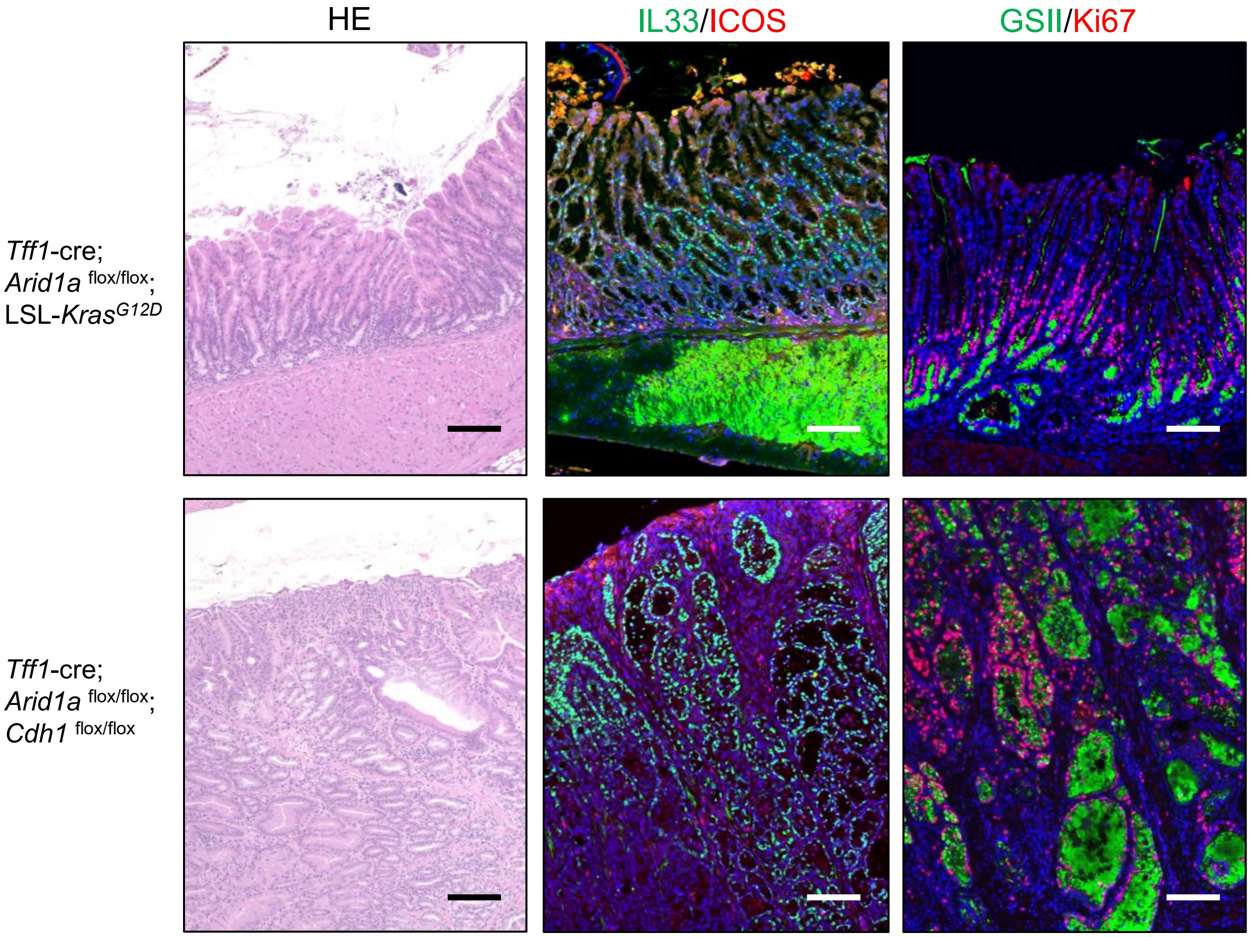

HE, GSII/Ki67, and IL33/ICOS staining in *Tff1*-Cre; *Arid1a*$^{flox/flox}$; LSL-*Kras*^{G12D} (top) and *Tff1*-Cre; *Arid1a*$^{flox/flox}$; *Cdh1*$^{flox/flox}$ mice (bottom) (independent repeats, n = 4). Scale bars represent 100 μm.

Supplementary Figure 12. Inhibition of type 2 immunity suppresses mutant *Arid1a*-dependent gastric tumorigenesis, related to Figure 6.

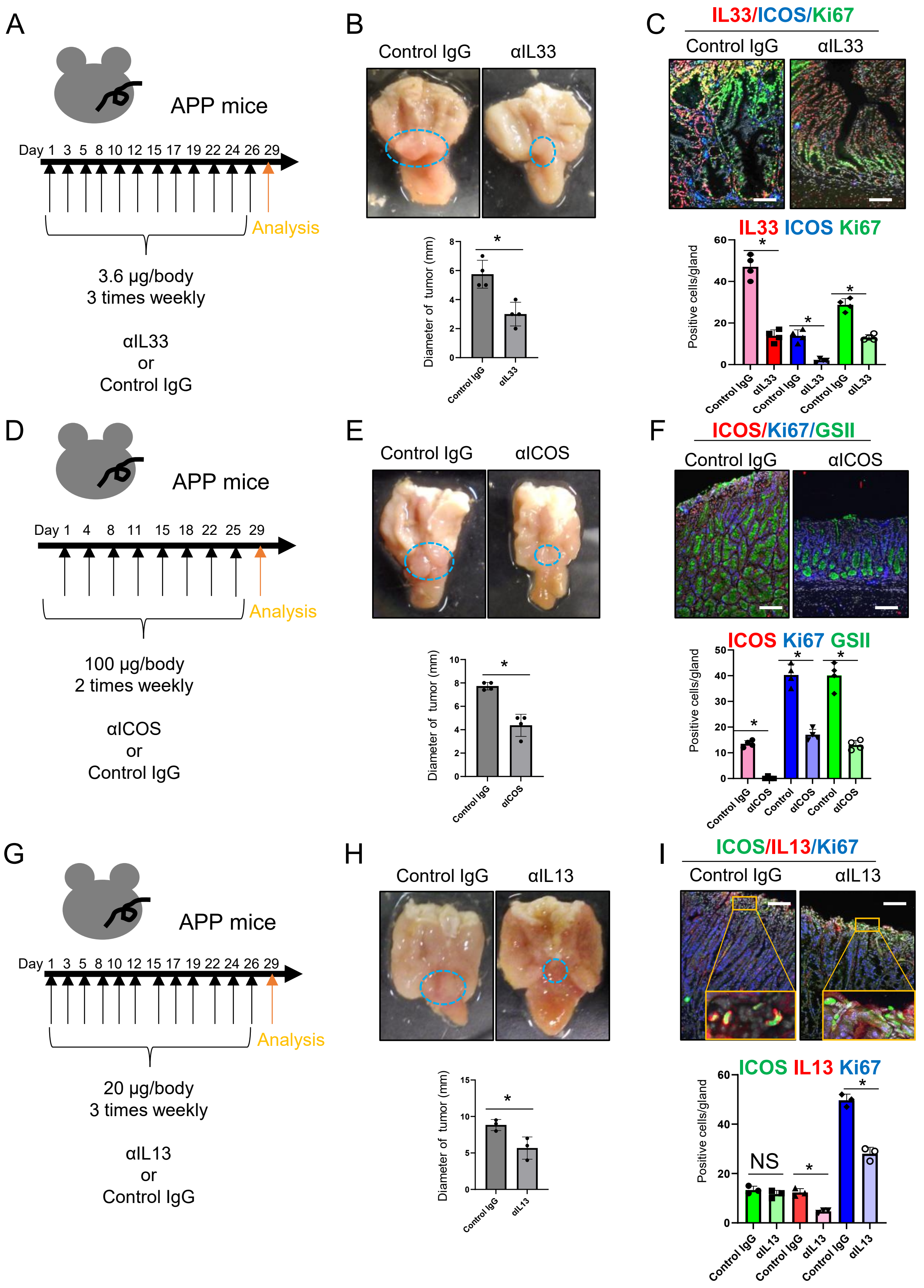

(A-C) Treatment with neutralizing antibody against IL33 in APP mice. (A) Schema of the treatment procedure; (B) gross findings. The diameter of the tumors (n=4/group) was quantified. (C) IL33 (red)/ICOS (blue)/Ki67 (green) staining in the control and treatment group. Numbers of cells expressing IL33 (pink/red), ICOS (blue/light blue), and Ki67 (green/light green) are quantified and shown at the bottom (n=4/group). The p-value was calculated using a t-test.

(D-F) Treatment with neutralizing antibody against ICOS in APP mice. (D) Schema of the treatment procedure; (E) gross findings. The diameter of the tumors (n=4/group) was quantified. (F) ICOS (red)/Ki67 (blue)/GSII (green) staining in the control and treatment group. Numbers of cells expressing IL33 (pink/red), ICOS (blue/light blue), and Ki67 (green/light green) are quantified and shown at the bottom (n=4/group). The p-value was calculated using a t-test.

(G-I) Treatment with neutralizing antibody against IL13 in APP mice. (G) Schema of the treatment procedure; (H) gross findings. The diameter of the tumors (n=3) was quantified. (I) IL13 (red)/Ki67 (blue)/ICOS (green) staining in control and treatment group. Numbers of cells expressing IL33 (pink/red), ICOS (blue/light blue), and Ki67 (green/light green) are quantified and shown at the bottom(n=3/group). The p-value was calculated using a t-test.

Mean ± S.E.M. *P < .05.

Supplementary Figure 13. Characterization of APP (Cre) organoids and xenografts, related to Figure 6.

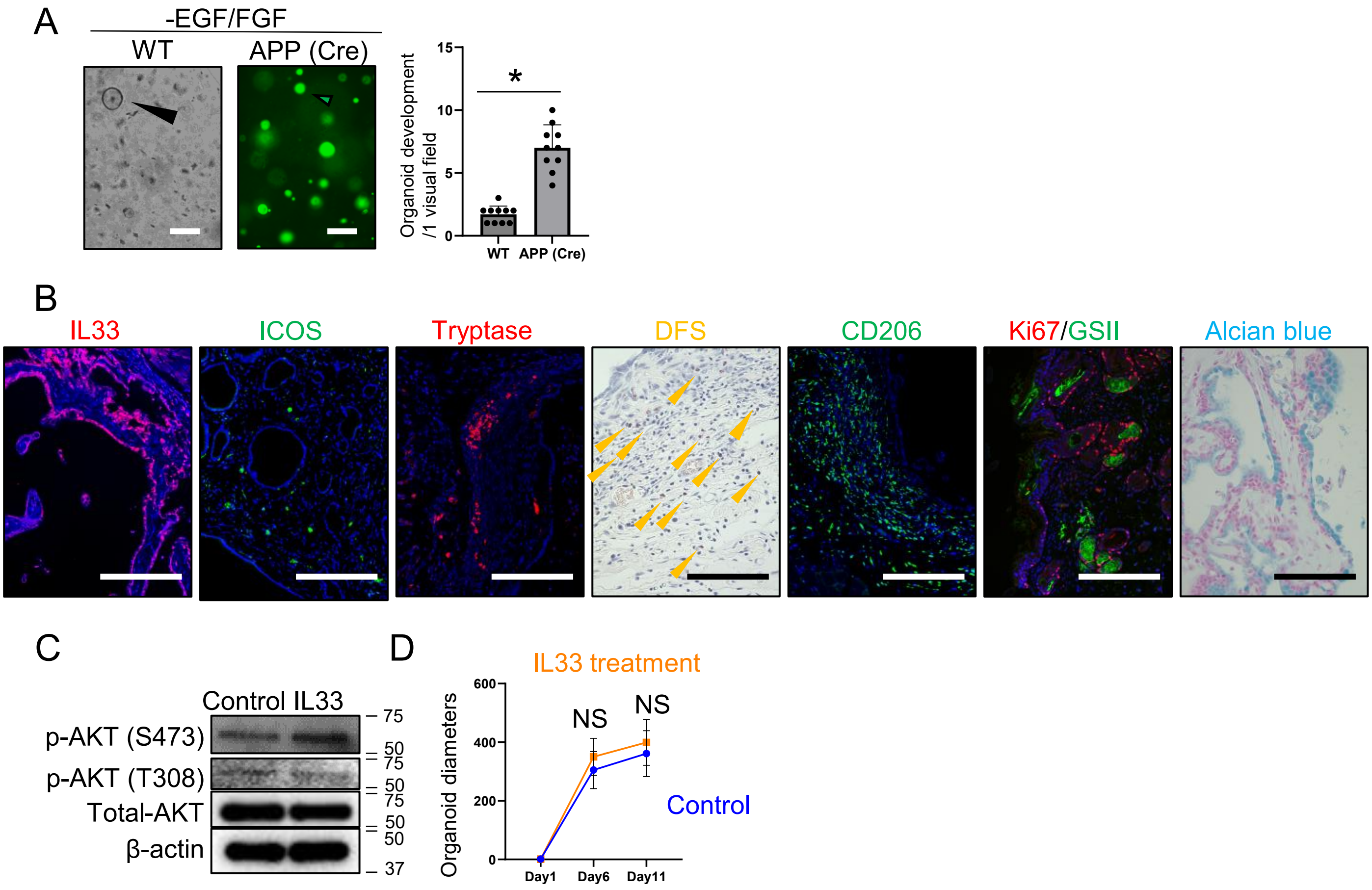

(A) WT and APP (Cre) organoid development in a medium without EGF/FGF reagents. Organoid development was quantified in each visual field (n=10/group). The p-value was calculated using a t-test.

(B) IL33 (red), ICOS (green) , Tryptase (red), DFS (brown), CD206 (green), Ki67 (red)/GSII (green), and Alcian blue staining of APP (Cre) xenografts (independent repeats, n = 9).

(C, D) IL33 administration on WT organoids. (C) Western blots of p-AKT (S473), p-AKT (T308), total-AKT, and β-actin are shown (independent repeats, n = 3). (D) Average organoid diameters were quantified (n = 20/group). The p-value was calculated using a t-test.

Mean ± S.E.M. *P < .05.

Supplementary Figure 14. TCGA database analysis for *ARID1A*-mutated or EBV-associated GCs, related to Figure 8.

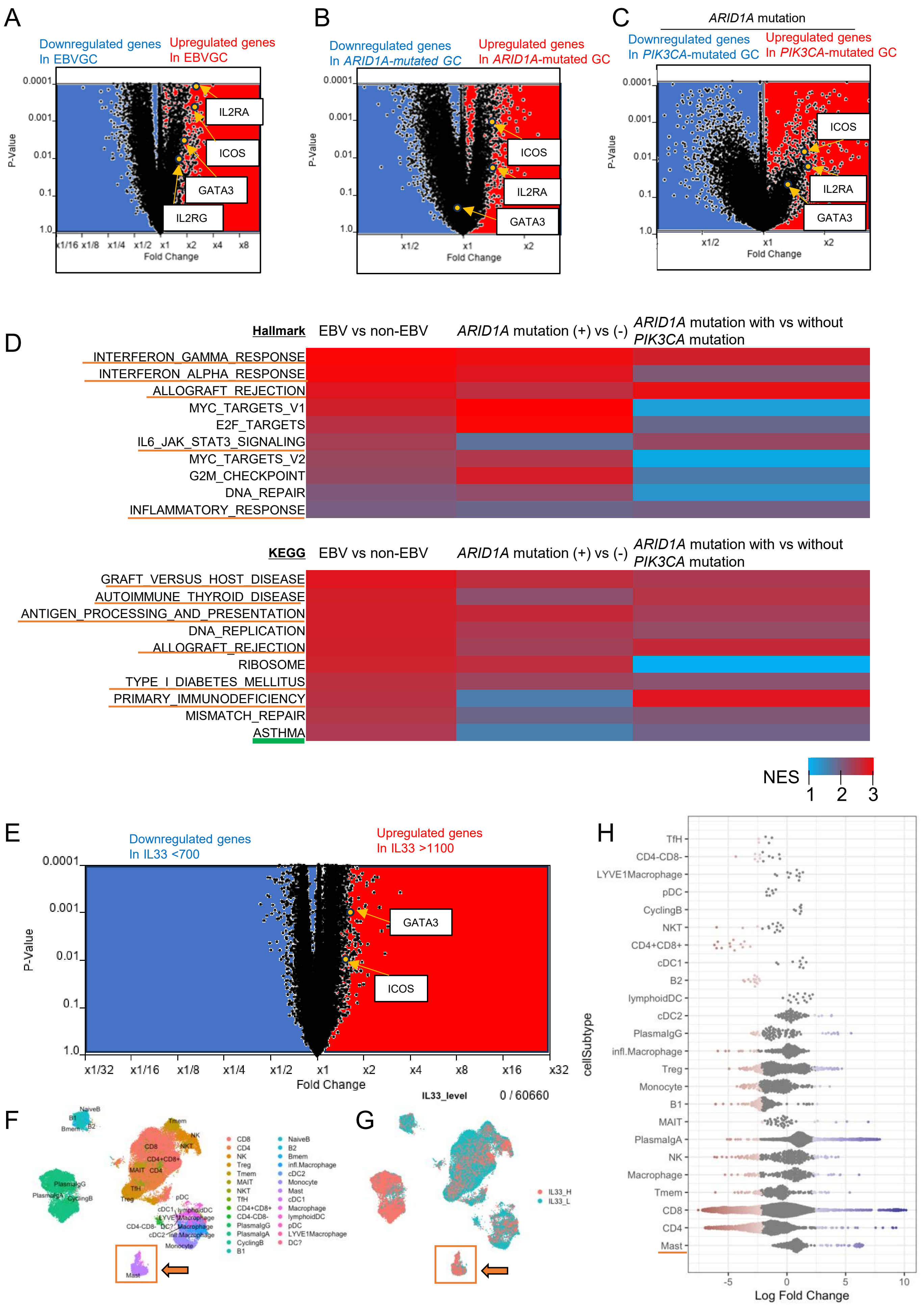

(A-D) Comparison GCs between (A) Epstein–Barr virus (EBV)-associated and non-associated cases (n = 24 vs 241), as well as (B)mutated and non-mutated *ARID1A* cases in total GCs (n = 84 vs 176) and (C) mutated and non-mutated *PIK3CA* cases with *ARID1A* mutation (n = 37 vs 47), from Cancer Genome Atlas data. (A-C) Volcano plots of DEGs are shown. Upregulated ILC2 markers are indicated by arrows. (D) Gene set enrichment analysis with Hallmark and Kegg gene sets.

(E) Comparisons between the top 25% and bottom 25% GCs based on IL33 expression (n = 66 vs 66). Volcano plot of DEGs is shown. Upregulated ILC2 markers are indicated by arrows.

(F) UMAP plot with clustering results from human gastric cancer tissues (GSE183904). (G) The same UMAP plot as in the panel (F) stratified on the basis of IL33 expression levels. (H) Differential abundance analysis between IL33-high and IL33-low samples from the same single cell RNA-seq data as in panels (F) and (G). Each dot is a neighborhood of cells calculated using miloR. Neighborhoods that reach significance (spatial FDR <0.1) are colored by log fold-change..

Supplementary Figure 15. Schema of adenovirus vectors, related to STAR★ Methods.

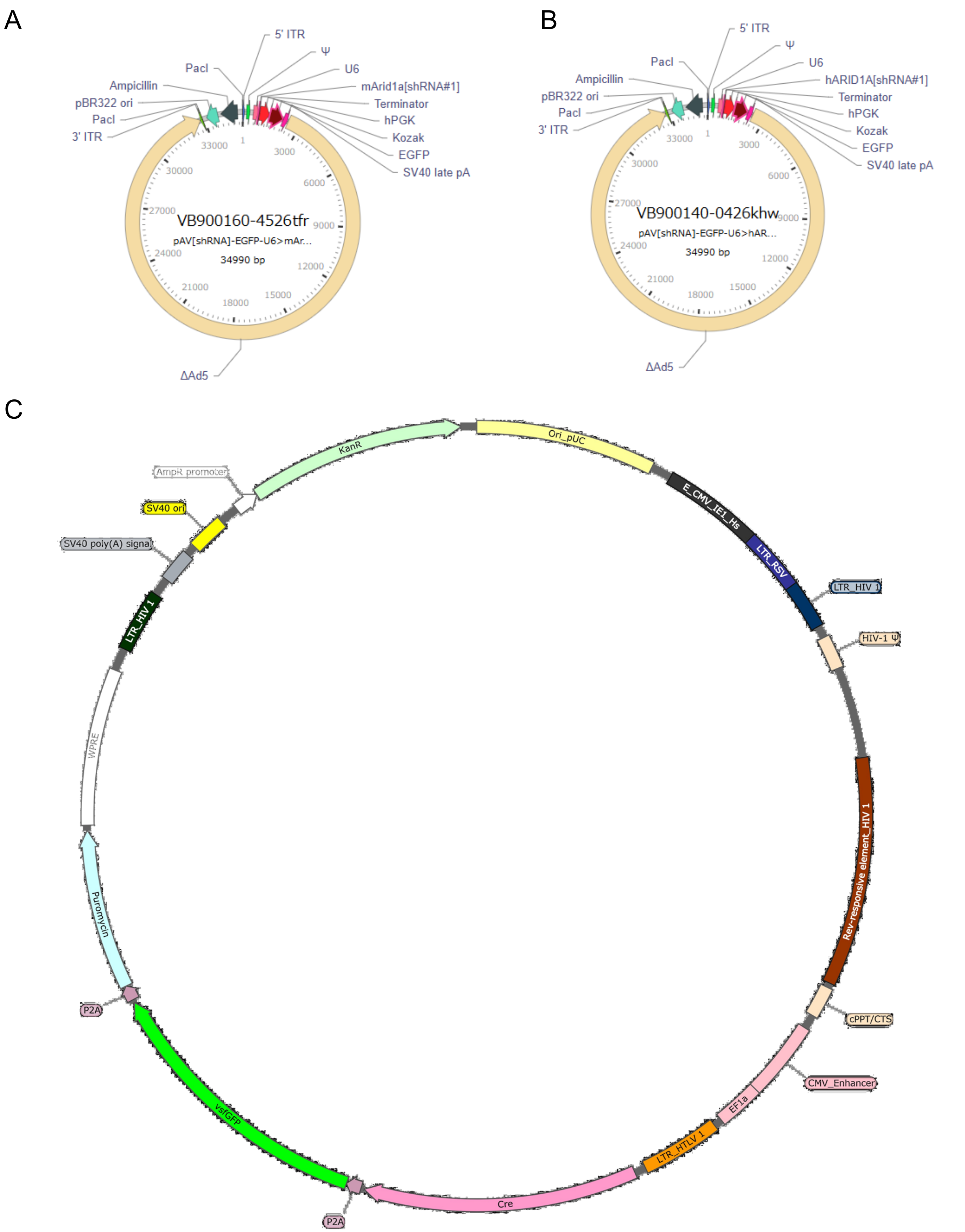

(A) pAV[shRNA]-EGFP-U6>mArid1a[shRNA#1]
(B) pAV[shRNA]-EGFP-U6>hARID1A[shRNA#1]
(C) pLV[Exp]-EGFP:T2A:Puro-EF1A>mCRE
